# Immunotherapy Bridge 2016 and Melanoma Bridge 2016: meeting abstracts

**DOI:** 10.1186/s12967-016-1095-2

**Published:** 2017-01-16

**Authors:** Vera Hirsh, Sandro Pignata, Melissa Bersanelli, Letizia Gnetti, Cinzia Azzoni, Lorena Bottarelli, Donatello Gasparro, Francesco Leonardi, Enrico Maria Silini, Sebastiano Buti, Erik Wennerberg, Aranzazu Mediero, Bruce Cronstein, Silvia Formenti, Sandra Demaria, Claire Vanpouille-Box, Karsten Pilones, Nils Rudqvist, Julie Diamond, Silvia Formenti, Sandra Demaria, Zachary S. Morris, Emily I. Guy, David M. Francis, Monica M. Gressett, Eric A. Armstrong, Shyhmin Huang, Stephen D. Gilles, Alan J. Korman, Jacquelyn A. Hank, Anna Hoefges, Alexander L. Rakhmilevich, Paul M. Harari, Paul M. Sondel, Yared Hailemichael, Willem W. Overwijk, Per thor Straten, Alessandro Lugli, Heather Dawson, Annika Blank, Inti Zlobec, Luigi Fattore, Susan Costantini, Mario Acunzo, Giulia Romano, Giovanni Nigita, Alessandro Laganà, Debora Malpicci, Ciro Francesco Ruggiero, Maria Elena Pisanu, Alessia Noto, Claudia De Vitis, Carlo Maria Croce, Paolo Antonio Ascierto, Rita Mancini, Gennaro Ciliberto, Michael Postow, Jason Luke, David Stroncek, Luciano Castiello, Wenjing Chen, Ping Jin, Jiaqiang Ren, Marianna Sabatino, Soldano Ferrone, Connie PM Duong, Marie Vetizou, Laurence Zitvogel, Maria Elena Pisanu, Alessia Noto, Luigi Fattore, Debora Malpicci, Gennaro Ciliberto, Rita Mancini, Marcella Occelli, Carolina Cauchi, Grazia Sciancalepore, Cristiana Lo Nigro, Michela Rovera, Chiara Varamo, Daniela Vivenza, Zelda Seia, Stefania Palazzini, Fabiana Errico, Davide Basso, Laura Quaranta, Giuseppe Forte, Fulvio Lavagna, Silvia Violante, Paolo Bosio, Laura Lattanzio, Marco Carlo Merlano, Duane Moogk, Shi Zhong, Zhiya Yu, Ivan Liadi, William Rittase, Victoria Fang, Janna Dougherty, Arianne Perez-Garcia, Iman Osman, Cheng Zhu, Navin Varadarajan, Nicholas P. Restifo, Alan Frey, Michelle Krogsgaard, Timea Balatoni, Anita Moho, Timea Sebestyén, Anita Varga, Judit Oláh, Zsuzsanna Lengyel, Gabriella Emri, Gabriella Liszkay, Andrea Ladányi, Beatrice Polini, Stefano Fogli, Sara Carpi, Barbara Pardini, Alessio Naccarati, Nevio Dubbini, Maria Cristina Breschi, Antonella Romanini, Paola Nieri, Francesca Morgese, Davide Soldato, Silvia Pagliaretta, Riccardo Giampieri, Donatella Brancorsini, Silvia Rinaldi, Mariangela Torniai, Anna Campanati, Giulia Ganzetti, Annamaria Offidani, Alfredo Giacchetti, Giuseppe Ricotti, Agnese Savini, Azzurra Onofri, Francesca Bianchi, Rossana Berardi, Giovanna Galdo, Gianfranco Orlandino, Salvatore Serio, Domenico Massariello, Tommaso Fabrizio, Valentina Montagnani, Matteo Benelli, Alessandro Apollo, Chiara Pescucci, Danilo Licastro, Carmelo Urso, Gianni Gerlini, Lorenzo Borgognoni, Lucio Luzzatto, Barbara Stecca, Elisabetta Gambale, Camilla Tinari, Alberto Quinzii, Alessio Cortellini, Consiglia Carella, Michele De Tursi, Adele Emanuela De Francesco, Mariarosanna De Fina, Maria Cristina Zito, Maria Dezia Bisceglia, Stefania Esposito, Giuseppina Fersini, Silvana Morello, Claudia Sorrentino, Aldo Pinto, Antonella Di Sarno, Antonella Bianco, Carmine D’Aniello, Francesca Andreozzi, Lucia Festina, Vito Vanella, Paolo Antonio Ascierto, Vincenzo Montesarchio, Beatrix Kotlan, Maria Godeny, Farkas Emil, Laszlo Toth, Szabolcs Horvath, Klara Eles, Timea Balatoni, Akos Savolt, Andras Szollar, Miklós Kasler, Gabriella Liszkay, Daniel Yiu, Fabio Grizzi, Federica Patrinicola, Maurizio Chiriva-Internati, Stefania Motta, Marcello Monti, Lavinia Benini, Stefano Ugel, Sara Cingarlini, Alessandra Fiore, Elisabetta Grego, Giampaolo Tortora, Vincenzo Bronte, Luca Tondulli, Gianluca Di Monta, Corrado Caracò, Ugo Marone, Lucia Festino, Paolo Antonio Ascierto, Nicola Mozzillo

**Affiliations:** 1 McGill University Health Centre Cedars Cancer Centre, Montreal, QC Canada; 2 Dipartimento Uro-Ginecologico S.C. Oncologia Medica Uro-Ginecologica, Istituto Nazionale Tumori Fondazione “G. Pascale”, Napoli, Italy; 3 Medical Oncology, University Hospital of Parma, Via Gramsci 14, 43126 Parma, PR Italy; 4 Anatomic Pathology Unit, University Hospital of Parma, Via Gramsci 14, 43126 Parma, PR Italy; 5 Weill Cornell Medicine, Cornell University, New York, NY USA; 6 New York University Langone Medical Center, New York, NY USA; 7 Department of Radiation Oncology, Weill Cornell Medical College, New York, NY USA; 8 Dept. of Human Oncology, School of Medicine and Public Health, University of Wisconsin, Madison, WI USA; 9 Provenance Biopharmaceuticals, Carlisle, MA USA; 10 Bristol-Myers Squibb Company, Redwood City, CA USA; 11 Department of Melanoma Medical Oncology, The University of Texas MD Anderson Cancer Center, Houston, TX USA; 12 Department of Hematology, Center for Cancer Immune Therapy (CCIT), University Hospital Herlev, Copenhagen, Denmark; 13 Department of Immunology and Microbiology, University of Copenhagen, Copenhagen, Denmark; 14 Institute of Pathology, University of Bern, Bern, Switzerland; 15 Istituto Nazionale per lo Studio e la Cura dei Tumori “Fondazione G. Pascale”, Naples, Italy; 16 Department of Molecular Virology, Immunology and Medical Genetics, The Ohio State University Comprehensive Cancer Center, Columbus, OH USA; 17 Department of Genetics and Genomic Sciences, Icahn School of Medicine at Mount Sinai, New York, NY USA; 18 Dipartimento di Medicina Sperimentale e Clinica, Università degli Studi di Catanzaro “Magna Graecia”, Catanzaro, Italy; 19 Dipartimento di Medicina Clinica e Molecolare, Sapienza Università di Roma, Rome, Italy; 20 Memorial Sloan Kettering Cancer Center, Rockville Centre, New York, NY USA; 21 University of Chicago Medicine, Chicago, Illinois USA; 22 Cell Processing Section, Department of Transfusion Medicine Clinical Center, NIH, Bethesda, MD USA; 23 Department of Surgery, Massachusetts General Hospital, Harvard Medical School, Boston, MA USA; 24 Institut de Cancérologie Gustave Roussy Cancer Campus (GRCC), Villejuif, France; 25 INSERM Unit U1015, Villejuif, France; 26 Université Paris Sud, Université Paris-Saclay Faculté de Médecine, Le Kremlin Bicêtre, France; 27 Center of Clinical Investigations in Biotherapies of Cancer 507, Villejuif, France; 28 Dipartimento di Chirurgia “P. Valdoni”, Sapienza Università di Roma, Rome, Italy; 29 Oncologia MedicaAzienda Ospedaliera Santa Croce e Carle, Cuneo, Italy; 30 Anatomia Patologica Azienda Ospedaliera Santa Croce e Carle, Cuneo, Italy; 31 CASAzienda Ospedaliera Santa Croce e Carle, Cuneo, Italy; 32 LILT, Cuneo, Italy; 33 SS Day Surgery Azienda Ospedaliera Santa Croce e Carle, Cuneo, Italy; 34 Chirurgia GeneraleAzienda Ospedaliera Santa Croce e Carle, Cuneo, Italy; 35 School of Medicine, Laura and Isaac Perlmutter Cancer Center, New York University, New York, NY 10016 USA; 36 Life Sciences Center, Xiangxue Pharmaceutical Co., Ltd., Guangzhou, China; 37 Center for Cancer Research, National Cancer Institute, US National Institutes of Health, Bethesda, MD 20892 USA; 38 Department of Chemical and Biomolecular Engineering, University of Houston, Houston, TX 77004 USA; 39 George W. Woodruff School of Mechanical Engineering, Georgia Institute of Technology, Atlanta, GA 30332-0405 USA; 40 NYU Medical Scientist Training Program (MSTP), New York, NY 10016 USA; 41 Kite Pharma Santa, Monica, CA 90404 USA; 42 Ronald Perelman Department of Dermatology, NYU School of Medicine, New York, NY 10016 USA; 43 Department of Cell Biology, School of Medicine, New York University, New York, NY 10016 USA; 44 Department of Pathology, School of Medicine, New York University, New York, NY 10016 USA; 45 Department of Oncodermatology, National Institute of Oncology, Budapest, Hungary; 46 1st Institute of Pathology and Experimental Cancer Research, Semmelweis University, Budapest, Hungary; 47 Department of Pathology, St. John’s Hospital, Budapest, Hungary; 48 Department of Dermatology and Allergology, Albert Szent-Györgyi Medical Center, University of Szeged, Szeged, Hungary; 49 Department of Dermatology, Venerology and Oncodermatology, University of Pécs, Pécs, Hungary; 50 Department of Dermatology, University of Debrecen, Debrecen, Hungary; 51 Department of Surgical and Molecular Pathology, National Institute of Oncology, Budapest, Hungary; 52 Department of Pharmacy, University of Pisa, Pisa, Italy; 53 Human genetic foundation (HUGEF), Torino, Italy; 54 Department of Medical Oncology, University Hospital of Pisa, Pisa, Italy; 55 Medical Oncology, Università Politecnica delle Marche, Azienda Ospedaliero-Universitaria Ospedali Riuniti Umberto I-GM Lancisi-G Salesi, Ancona, Italy; 56 Section of Pathological Anatomy and Histopathology, Deparment of Neuroscience, Università Politecnica delle MarcheAzienda Ospedaliero-Universitaria Ospedali Riuniti Umberto I-GM Lancisi-G Salesi, Ancona, Italy; 57 Dermatology Clinic Università Politecnica delle Marche Azienda Ospedaliero-Universitaria Ospedali Riuniti Umberto I-GM Lancisi-G Salesi, Ancona, Italy; 58 U.O. DermatologiaI NRCA/IRCCS, Ancona, Italy; 59 Plastic Surgery Unit, IRCCS-CROB, Rionero in Vulture, PZ Italy; 60 Core Research Laboratory-Istituto Toscano Tumori, Florence, Italy; 61 Diagnostic Genetics Unit, Careggi University Hospital, Florence, Italy; 62 CBM Genomics Area Science Park Basovizza, Trieste, Italy; 63 Anatomic Pathology UnitDermatopathology Section, S.M. Annunziata Hospital, Florence, Italy; 64 Plastic Surgery Unit, S.M. Annunziata Hospital, Florence, Italy; 65 Department of Medical, Oral and Biotechnological Sciences“G. D’Annunzio”, University of Chieti-Pescara, Chieti, Italy; 66 Unit of Endocrinology, Department of Medicine and Sciences of Aging Ce.S.I.-Me.T.“G.D’ Annunzio”, University of Chieti-Pescara, Chieti, Italy; 67 Medical Oncology, San Salvatore Hospital, L’Aquila, Italy; 68 Department of Biotechnological and Applied Clinical Sciences, University of L’Aquila, L’Aquila, Italy; 69 Oncology Unit, “SS. Annunziata” Hospital, Chieti, Italy; 70 Mater Domini University Hospital, Pharmacy Unit, Catanzaro, Italy; 71 Settore Politiche del Farmaco, Dipartimento Tutela della Salute, Catanzaro, Italy; 72 Department of Pharmacy, University of Salerno, Fisciano, 84084 Salerno, Italy; 73 UOC of Oncology A.O.R.N. dei Colli, Monaldi Hospital, Naples, Italy; 74 IRCCS Pascale, Naples, Italy; 75 Molecular Immunology and Toxicology, National Institute of Oncology, Budapest, Hungary; 76 Radiological Department, National Institute of Oncology, Budapest, Hungary; 77 Center of Oncosurgery, National Institute of Oncology, Budapest, Hungary; 78 Center of Surgical and Molecular Tumorpathology, National Institute of Oncology, Budapest, Hungary; 79 Board of Directors, National Institute of Oncology, Budapest, Hungary; 80 Humanitas University, Rozzano, Milan, Italy; 81 Department of Immunology and Inflammation, Humanitas Clinical and Research Hospital, Rozzano, Milan, Italy; 82 Kiromic LLC, Houston, TX USA; 83 University of Milan, Milan, Italy; 84 Department of Immunology, Policlinico G.B. Rossi Hospital, Verona, Italy; 85 Department of Medical Oncology, Policlinico G.B. Rossi Hospital, Verona, Italy; 86 Dipartimento Melanoma, Tessuti molli, Muscolo-Scheletrico e Testa-Collo Oncologia Medica Melanoma Immunoterapia Oncologica e Terapie Innovative Istituto Nazionale Tumori “G. Pascale”, Via M. Semmola, 80131 Naples, Italy

## Immunotherapy **Bridge 2016**: Keynote speaker presentations

### Immunotherapy beyond melanoma

#### K1 Checkpoint inhibitors anti PD1/PDL1 in metastatic NSCLC

##### Vera Hirsh

###### Cedars Cancer Centre, McGill University Health Centre, Montreal, QC, Canada

####### **Correspondence:** Vera Hirsh - vera.hirsh@muhc.mcgill.ca

*Journal of Translational Medicine* 2016, **15(Suppl 1)**:K1

**Background:** NSCLC remains a challenging disease to treat, especially because the majority of patients present with advanced disease [1]. For many of these patients the standard first-line therapy is platinum-based chemotherapy, which can prolong survival by 8–12 months in some patients and also improve disease-related symptoms [2]. The chemotherapy treatments are frequently poorly tolerated. Treatment choices following chemotherapy are limited, approved options include docetaxel, pemetrexed and erlotinib. Recent developments with immunotherapies and approval of agents such as nivolumab and pembrolizumab [3, 4] are exciting. Other immunotherapy agents such as darvolumab, atezolizumab and avelumab are also investigated and show good results.

**Materials and methods:** Literature was reviewed about checkpoint inhibitors in metastatic NSCLC. Both the efficacy, patient reported outcomes and adverse events will be reported, with emphasis on the results from phase 3 trials.

**Results:** Checkpoint inhibitors in development are ipilimumab and tremelimumab, they block CTLA4. Nivolumab and pembrolizumab are blocking binding of PD1 to PDL1 and PDL2. Atezolizumab and durvalumab are blocking binding of PDL1 to PD1 and CD 80. Avelumab is in early stages of research. Nivolumab trials CheckMate 017 [5] and 057 in second-line phase 3 trials and ChecMate 026 in 1st-line monotharapy vs SOC phase 3 trial were reported. In second-line nivolumab showed superiority over docetaxel in progression free survival (PFS) 3.5 vs 2.8 months, HR = 0.62, p- 0.004, mOS = 9.2 vs 6 months independent to PDL1 expression in squamous histology. Adverse events were rare and manageable. Combination of nivolumab with chemotherapy and with erlotinib are being investigated. Pembrolizumab monotherapy showed responses which exceeded one year, median PFS = 6.3 months, especially high tumor proportion score (TPS) ≥50%, representing 23% of the screened NSCLC population. In pembrolizumab vs docetaxel, Keynote 010, median OS with pembrolizumab 2 mg/kg and at least 50% PDL1 TPS was 14.9 months vs 8.2 months on docetaxel. Adverse events were less common on pembrolizumab. PDL1 expression ≥50% correlated with improved RR,PFS and OS regardless of histology. Smoking status was associated with increased RR. Keynote 024 = pembrolizumab vs chemotherapy showed superiority in phase 3, chemonaive patients of pembrolizumab in OS. (=Press release only) Atezolizumab vs docetaxel, phase 2 randomized trial [6] POPLAR showed improvement in OS (HR 0.69 vs 0.73) in ITT population. Duration of response of 18.6 months compared to 7.2 months with docetaxel and well tolerated safety profile.OAK trial = phase 3 is ongoing. Durvalumab with trmelimumab phase 3 trial results are pending.

**Conclusion:** The results of immunotherapy trials are encouraging, both from the point of improved efficacy and their tolerability. Combination treatments with immunotherapy may further improve the efficacy and prolong the lives of the patients with metastatic NSCLC. These combination treatments will replace eventually the platinum doublets in first-line treatment of metastatic NSCLC

**References**

1. Saintigny P, Burger JA. Recent advances in non-small cell lung cancer biology and clinical management. Disov Med. 2012;13(71):287–97.

2. Leighl NB. Treatment paradigms for patients with metastatic non-small-cell lung cancer: first-, second-, and third-line. Curr Oncol. 2012;19(1):S52–S8.

3. Borghaei H, Paz-Ares L, Horn L et al. Nivolumab vs docetaxel in advanced nonsquamous non-small-cell lung cancer. N Engl J Med. 2015;373(17):627–39.

4. Herbst RS, BAAS P, Kim DW et al. Pembrolizumab versus docetaxel for previously treated, PD-L1-positive, advanced non-small-cell lung cancer (KEYNOTE-010): a randomized controlled trial. Lancet. 2015;387:1540–50.

5. Brahmer J, Reckamp KL, Baas P et al. Nivolumab vs docetaxel in advanced squamous-cell non-small-cell lung cancer. N Engl J Med. 2015;373(2):123–35.

6. Fehrenbacker L, Spira A, Ballinger M et al. Atezolizumab vs docetaxel for patients with previously treated non-small-cell lung cancer (POPLAR): a multicentre, open-label, phase 2 randomised controlled trial. Lancet. 2016.

### Immunotherapy **in oncology**: data from clinical trial

#### K2 Immunotherapy in ovarian and endometrial cancer

##### Sandro Pignata

###### Dipartimento Uro-Ginecologico S.C. Oncologia Medica Uro-Ginecologica, Istituto Nazionale Tumori Fondazione “G. Pascale”, Napoli, Italy

####### **Correspondence:** Sandro Pignata - s.pignata@istitutotumori.na.it

*Journal of Translational Medicine* 2016, **15(Suppl 1)**:K2

Ovarian (OC) and Endometrial Cancer (EC) are still a challenge for gynecological oncologists because the treatment of the advanced disease remains an unmet need for patients. Some literature data confirmed the hypothesis of an immunogenic profile of both tumors. Some evidences showed an high mutational load and a unique mutational signature of BRCA1/2 mutated high grade serous ovarian cancers (HGSOCs) that probably harbor more tumor-specific neoantigens and increased TILs and PD-1/PD-L1 expression [1]. The Cancer Genome Atlas Research Network (TCGA) recently provided a comprehensive genomic and transcriptomic analysis of OC and EC offering a new classification of those diseases, based on genetic features. Two analysis of gene profiling of high grade serous OC identified immunoreactive tumors (with a characteristic gene signature) that seemed to be related to a better prognosis, compared to the other subgroups [2, 3]. Other similar analysis on EC tumors, identified two among four subgroups (the polymerase epsilon [POLE]-ultramutated and the microsatellite instability [MSI]-hypermutated), that seem to present an enhanced immune microenvironment and a high mutation burden [4, 5]. Immune checkpoint inhibitors (e.g., anti-PD-1 and anti-PD-L1 antibodies) have demonstrated remarkable efficacy against hypermutated cancers such as melanomas and lung carcinomas. One explanation for this effect is that hypermutated lesions harbor more tumor-specific neoantigens that stimulate recruitment of an increased number of tumor-infiltrating lymphocytes (TILs), which is counterbalanced by overexpression of immune checkpoints such as PD-1 or PD-L1. Encouraging data support the relationship between the over-expression of the PD-1/PD-L1 pathway in OCs and ECs with a higher genomic instability, and the activity of immunecheckpoint inhibitors [6, 7]. Those evidences suggest a rationale for testing the PD-1/PD-L1 immunotherapy in these cancer subgroups.

**References**

1. Strickland KC, Howitt BE, Shukla SA, et al. Oncotarget. 2016;7(12):13587–98.

2. The Cancer Genome Atlas Research Network. Nature. 2011;474(7353):609–15.

3. Charlie Gourley et al. ASCO meeting. 2014.

4. R. Murali, R.A. Soslow, B. Weigelt Classification of endometrial carcinoma: more than two types Lancet Oncol. 2014;15(7):e268–78.

5. Meng B, Hoang LN, McIntyre JB et al. POLE exonuclease domain mutation predicts long progression-free survival in grade 3 endometrioid carcinoma of the endometrium. Gynecol Oncol. 2014;134(1):15–9.

6. Zhang L, Conejo-Garcia JR, Katsaros D et al. Intratumoral T cells, recurrence, and survival in epithelial ovarian cancer. N Engl J Med. 2003;348(3):203–13.

7. Howitt BE, Shukla SA, Sholl LM et al. Association of polymerase e-mutated and microsatellite-instable endometrial cancers with neoantigen load, number of tumor-infiltrating lymphocytes, and expression of PD-1 and PD-L1. JAMA Oncol. 2015;1(9):1319–23.

## Immunotherapy Bridge 2016: oral presentations

### Immunotherapy beyond melanoma

#### O1 LOH as “the missing instability” potentially underlying the tumor immunogenicity: on the trails of a correlation between fractional allelic loss and response to nivolumab in renal cancer

##### Melissa Bersanelli^1^, Letizia Gnetti^2^, Cinzia Azzoni^2^, Lorena Bottarelli^2^, Donatello Gasparro^1^, Francesco Leonardi^1^, Enrico Maria Silini^2^, Sebastiano Buti^1^

###### ^1^University Hospital of Parma, Medical Oncology, via Gramsci 14, 43126, Parma, PR, Italy, ^2^University Hospital of Parma, Anatomic Pathology Unit, Via Gramsci 14, 43126, Parma, PR, Italy

####### **Correspondence:** Melissa Bersanelli - bersamel@libero.it

*Journal of Translational Medicine* 2016, **15(Suppl 1)**:O1

**Background:** Microsatellite instable (MSI) phenotype, as expression of mismatch-repair deficiency, characterizes tumors with high mutational load and consequently increased production of immunogenic neoantigens [1]. These characteristics have been correlated with susceptibility to immune checkpoint inhibitors (CKI) [2]. Nevertheless, MSI is rare (34%) in renal cell carcinoma (RCC), although CKI are effective in a significant fraction of advanced tumors. We evaluated the hypothesis that loss of heterozygosity (LOH) of relevant tumor suppressor genes (TSG) might provide a measure of tumor immunogenicity, contributing as “the missing instability” to a high fractional allele loss (FAL) potentially underlying the rate of disease control obtained with CKI. We preliminary report the possible predictive value of LOH at different loci on 3p in patients with advanced RCC treated with the anti-PD-1 Nivolumab.

**Materials and methods:** Only patients with assessable response according to the immune-related radiologic criteria were included. After DNA extraction from FFPE tissues of nephrectomy specimens, LOH study was performed by PCR for the microsatellite markers D3S1481 (3p14.2, *FHIT* gene) and D3S1478 (3p21.321.2). Non-informative (NI) cases due to homozygosity were subsequently tested for D3S1621 (3p21.2, *LCTSGR1* gene). Non-informative cases for all loci were excluded. MSI was also assessed using two classical mononucleotide markers, BAT-25 and BAT-26.

**Results:** Nine RCC cases evaluable for response were analyzed. All of them had microsatellite stable phenotype (MSS). Five cases (45%) presented LOH: three only at the *FHIT* locus (D3S1481), two at both 3S1481 and D3S1478 loci. All these patients with LOH of at least one 3p locus had good disease control and clinical benefit with Nivolumab: two had partial response and three had stable disease (SD). The remaining four cases were negative for LOH at the informative loci: three of them were clearly non-responsive to Nivolumab (confirmed progression disease) and one had SD.

**Conclusion:** These preliminary results suggest that LOH at 3p loci correlates with good disease control with Nivolumab in advanced RCC patients. Our findings support the hypothesis that LOH at relevant genetic loci may provide a measure of mutational burden and of tumor immunogenicity and it could be used as predictive biomarker of response to CKI. Further analyses are planned with the aim to investigate other loci of key-TSG on 3p, 9p and 14p, evaluating the FAL index as potential more reliable predictive factor.

**References**

1. Voss ME, Hellmann MD, Chen Y, Gold TM, Lambert CR, VanAllen E, Choueiri TK, Charen AS, Motzer RJ. Mutation burden and tumor neoantigens in RCC patients treated with Nivolumab: J Clin Oncol. 2016,34(2). **(abstr 514)**.

2. Le DT, Uram JN, Wang H, Bartlett BR, Kemberling H, Eyring AD, Skora AD, Luber BS, Azad NS, Laheru D et al. PD-1 blockade in tumors with mismatch-repair deficiency. N Engl J Med. 2015;372(26):2509–20.

### Immunotherapy in oncology: data from clinical trial

#### O2 Adenosine generation limits the ability of radiation therapy to elicit anti-tumor immunity by hindering recruitment and activation of CD103^+^ DCs

##### Erik Wennerberg^1^, Aranzazu Mediero^2^, Bruce Cronstein^2^, Silvia Formenti^3^, Sandra Demaria^1^

###### ^1^Weill Cornell Medicine, New York, NY, USA, ^2^New York University Langone Medical Center, New York, NY, USA, ^3^Department of Radiation Oncology-Weill Cornell Medical College, New York, NY, USA

####### **Correspondence:** Erik Wennerberg - erw2010@med.cornell.edu

*Journal of Translational Medicine* 2016, **15(Suppl 1)**:O2

**Background:** Localized radiation therapy (RT) can act as a powerful adjuvant to immunotherapeutic strategies by triggering *de novo* anti-tumor immune responses to poorly immunogenic tumors. Radiation of tumor cells induces a dose-dependent release of ATP, a molecule that when released in the tumor microenvironment (TME) triggers recruitment and activation of dendritic cells (DCs), including CD103^+^ DCs recently identified as the key DC subset responsible for cross-presentation of tumor-derived antigens to CD8^+^ T cells [1,2]. However, rapid hydrolysis of extracellular ATP by ecto-enzymes CD39 and CD73 results in a local accumulation of immunosuppressive adenosine (ADO) that inhibits DC activation and CD8^+^ T cell effector functions and enhances regulatory T cells (Tregs). By blocking CD73 in irradiated tumors, we tested the hypothesis that ADO generation limits the ability of RT to trigger anti-tumor immunity.

**Materials and methods:** Wild type (WT) or BATF3^−/−^ mice (ablated development of CD8a^+^/CD103^+^ DCs) were inoculated s.c. with the poorly immunogenic breast cancer cell line TSA on day 0 and assigned to treatment with: (1) control Ab; (2) anti-CD73 (TY/23 Ab) (3) RT (20 Gy); (4) RT + TY/23. TY/23 (100 μg) was administered i.p. on day 11, 14, 17 and 20. RT was given locally to the tumor as single 20 Gy dose on day 12. On day 18, some tumors were harvested for flow cytometry analysis of DCs and T cells. Mice were monitored for tumor progression by caliper measurements.

**Results:** In irradiated but not sham-treated mice, anti-CD73 Ab resulted in increased infiltration of CD103^+^DCs (8.9 ± 2.6% of DCs in RT + anti-CD73 v. 3.5 ± 2.8% of DCs in RT, p < 0.05) expressing elevated levels of activation markers CD40 and CD86 compared to mice treated with RT alone. This change was associated with improved CD8^+^T cell/Treg ratio (5 ± 2.8 in RT + anti-CD73 v. 0.8 ± 0.2 in RT). Importantly, CD73 blockade had no effect by itself but improved significantly radiation-induced tumor control (Tumor size at day 57 post inoculation: 385 ± 525 mm^3^ in RT + anti-CD73 v. 1036 ± 727 mm^3^ in RT). Consistent with the hypothesis that CD103^+^ DCs are essential for anti-tumor responses, the therapeutic effect of RT + CD73 blockade was abrogated in BATF3^−/−^ mice.

**Conclusion:** Our data indicate that ADO generated following RT plays a key role in hindering development of anti-tumor immune responses and identify, as a mechanism of this effect, an abrogated infiltration and activation of CD103^+^ DCs. Blockade of ADO generation by anti-CD73 treatment constitutes a promising strategy to enhance radiation-induced anti-tumor immunity.

**References**

1. Golden EB, Frances D et al. Radiation fosters dose-dependent and chemotherapy-induced immunogenic cell death. Oncoimmunology. 2014;3:e28518.

2. Broz ML, Binnewies M et al. Dissecting the tumor myeloid compartment reveals rare activating antigen-presenting cells critical for T cell immunity. Cancer Cell. 2014;26(5):638–52.

## Melanoma Bridge 2016: Keynote speaker presentations

### Evolving topics in cancer immunotherapy

#### K3 Treatment-induced molecular pathways regulate the immunogenicity of radiotherapy

##### Claire Vanpouille-Box, Karsten Pilones, Nils Rudqvist, Julie Diamond, Silvia Formenti, Sandra Demaria

###### Weill Cornell Medicine, Cornell University, New York, NY, USA

####### **Correspondence:** Sandra Demaria - szd3005@med.cornell.edu

*Journal of Translational Medicine* 2016, **15(Suppl 1)**:K3

Elimination of virally-infected epithelial cells is mediated by CD8^+^ T cells and results in life-long protective immunity against reinfection. Similarly, clinical data have shown that CD8^+^ T cells mediate the rejection of solid tumors and can confer long-term protection from disease recurrence when their activity is unleashed by immune checkpoint inhibitors. Like viral proteins, mutated proteins expressed by an individual tumor are a source of powerful tumor-specific T cell epitopes. However, most of the cancer patients do not develop a sufficient number and repertoire of tumor-reactive T cells and are unresponsive to currently available immunotherapies. We have pioneered studies to explore the use of local tumor radiotherapy (RT) as a means to release tumor antigens in an immunogenic context and induce anti-tumor CD8 T cells. We have demonstrated that RT in combination with anti-CTLA-4 elicits powerful and durable anti-tumor responses in pre-clinical models and in at least some patients (1, 2). Mechanistically, we have found that in tumors treated with RT + anti-CTLA-4 there is a marked increase in the CD8/CD4 ratio paralleled by an increase in clonality of the T cell receptor (TCR) repertoire. Notably, unbiased clustering analysis of the TCR Vβ chain CDR3 sequences shows that there are unique clusters in each treatment group (RT alone, anti-CTLA-4 alone, RT + anti-CTLA-4) that are not seen in untreated tumors. Importantly, most of the TCRs seen in the combination group do not overlap the TCR present in each monotherapy group, indicating that a unique TCR repertoire is generated by RT + anti-CTLA-4. Studies to optimize the vaccination effect of RT show that canonical pathways activated during viral infection are induced by hypofractionated (8GYX3) but not high single dose (20 or 30 Gy) RT and explain the differential ability of these RT regimens to synergize with anti-CTLA-4 in generating anti-tumor T cells and achieving abscopal responses. Overall, radiation-induced cancer cell-intrinsic activation of interferon type I pathway is absolutely required for the generation of systemically effective and lasting anti-tumor immunity against poorly immunogenic tumors.

**References**

1. Vanpouille-Box C, Pilones KA, Wennerberg E, Formenti SC, Demaria S. In situ vaccination by radiotherapy to improve responses to anti-CTLA-4 treatment. Vaccine. 2015;33:7415–22.

2. Golden EB, Demaria S, Schiff PB, Chachoua A, Formenti SC. An abscopal response to radiation and ipilimumab in a patient with metastatic non-small cell lung cancer. Cancer Immunol Res. 2013;1:365–72.

#### K4 In situ tumor vaccination using local radiation therapy (RT) and intratumoral immunocytokine (IC)

##### Zachary S. Morris^1^, Emily I. Guy^1^, David M. Francis^1^, Monica M. Gressett^1^, Eric A. Armstrong^1^, Shyhmin Huang^1^, Stephen D. Gilles^2^, Alan J. Korman^3^, Jacquelyn A. Hank^1^, Anna Hoefges^1^, Alexander L. Rakhmilevich^1^, Paul M. Harari^1^, Paul M. Sondel^1^

###### ^1^Dept. of Human Oncology, University of Wisconsin School of Medicine and Public Health, Madison, WI, USA, ^2^Provenance Biopharmaceuticals, Carlisle, MA, USA, ^3^Bristol-Myers Squibb Company, Redwood City, CA, USA

####### **Correspondence:** Paul M. Sondel - pmsondel@humonc.wisc.edu

*Journal of Translational Medicine* 2016, **15(Suppl 1)**:K4

We have identified a cooperative interaction between local tumor RT (single fraction 12 Gy), intratumoral (IT) injection of IC hu14.18-IL2 (anti-GD2 hu14.18 mAb linked to IL2), and checkpoint blockade with anti-CTLA4 mAb. C57Bl/6 mice were implanted with 2 × 10^6^ B78 (GD2+) melanoma in one flank (1-tumor model). For mice bearing a single 5 week (w) 200 mm^3^ tumor, RT + IT-IC results in complete response (CR) in 71% of mice and a tumor-specific memory T cell response. In the 2-tumor model, mice received 2 × 10^6^ B78 3 weeks (w) after the initial tumor implantation, in the opposite flank, and was ~50 mm^3^ at 5w, when RT begins. Surprisingly, RT + IT-IC to the 1st ~200 mm^3^ tumor, but not to the 2nd tumor, did not enhance 1st tumor shrinkage compared to RT alone and had no effect on the 2nd tumor. The 2nd tumor caused immune suppression, preventing RT and IT-IC from eliminating the 1st tumor. This was tumor-specific, as local RT + IT-IC to the 1st tumor was still effective if the 2nd was the unrelated Panc02 tumor. Delivering RT to both the 1st + 2nd B78 tumors eliminated the inhibitory effect of the 2nd tumor, enabling IT-IC to the 1st tumor eradicated it in 64% of mice. Preliminary PCR and flow analyses showed Tregs are better depleted by 1st tumor RT in mice with 1 vs 2 tumors. In this 2 tumor model we combined RT + IT-IC of the 1st tumor with an IgG2a anti-CTLA-4 (that depletes Tregs); this rendered 60% of mice disease-free (durable CR of both the treated 1st and the untreated 2nd tumors). Overall, local RT + IT-IC can result in long-term tumor eradication of large tumors via adaptive immunity to the “in situ vaccine”, provided that Treg-associated immune suppression from distant tumor is eliminated by RT or Treg-depletion.

#### K5 Cancer vaccines in the era of checkpoint blockade

##### Yared Hailemichael, Willem W. Overwijk

###### Department of Melanoma Medical Oncology, The University of Texas MD Anderson Cancer Center, Houston, TX, USA

####### **Correspondence:** Willem W. Overwijk - woverwijk@mdanderson.org

*Journal of Translational Medicine* 2016, **15(Suppl 1)**:K5

Anti-cancer vaccination is a promising approach to increase the efficacy of checkpoint blockade therapies. There exists a broad range of vaccine formulations, each with their unique benefits and limitations. Clinically, the vaccine most commonly used in combination with checkpoint blockade is based on peptide antigen formulated in mineral oil, also known as incomplete Freund’s Adjuvant (IFA). However, the landmark FDA registration trial for anti-CTLA-4 therapy (Ipilimumab) revealed a complete lack of benefit of adding vaccination with gp100 peptide formulated in IFA, and in fact demonstrated a significant reduction in initial disease control. Upon modeling this combination therapy in mice, we found that gp100 peptide in IFA induces gp100-specific Teff which become sequestered at the vaccination site and cause local inflammation. The inflamed vaccination site subsequently also sequesters and destroys the systemic pool of anti-CTLA-4 induced Teff with specificities for tumor antigens other than gp100, reducing the anti-tumor efficacy of anti-CTLA-4 therapy. We identify key mechanistic mediators of T cell sequestration, as well as alternative vaccine approaches that do not induce these undesirable effects and instead potently synergize with anti-CTLA-4 and anti-PD-1 checkpoint blockade, causing markedly increased anti-tumor activity. These findings point the way to rational design and selection of cancer vaccine formulations that synergize with checkpoint blockade therapy.

#### K6 Therapy of solid cancers using T cells and running shoes

##### Per thor Straten^1,2^

###### ^1^Center for Cancer Immune Therapy (CCIT), Department of Hematology, University Hospital Herlev, Copenhagen, Denmark, ^2^Department of Immunology and Microbiology, University of Copenhagen, Copenhagen, Denmark

####### **Correspondence:** Per thor Straten - ptstraten@sund.ku.dk

*Journal of Translational Medicine* 2016, **15(Suppl 1)**:K6

Cells of the immune system, e.g., CD8 T cells and Natural killer (NK) cells are capable of recognizing and killing cancer cells. Moreover, several lines of evidence suggest that the naturally elicited anti-cancer immune responses lead to infiltration of immune cells into tumors and the presence of these cells have an impact on disease progression, i.e., overall survival of the patient. Harnessing of the immune system to combat cancer i.e., cancer immunotherapy, is revolutionizing treatment of patients today. To this end, administration of immune check inhibitory monoclonal antibodies is approved for the treatment of several solid cancer indications, and although predictive markers are still missing, patients with a brisk tumor infiltration of immune cells are more prone to respond to treatment. Moreover, immune cells infiltrating into the tumor can be directly utilized in therapy by administration of in vitro expanded tumor infiltrating lymphocytes (TIL). In our clinical trials based on TIL therapy we have experienced massive tumor regressions in melanoma patients, some of which experience potentially curative complete responses. The success of TIL therapy in melanoma, clearly builds on the presence of tumor specific T cells at the tumor site, and underscores the need to define ways as how to increase immune cell infiltration to the tumor site. Regular exercise reduces the risk of cancer and disease recurrence, by largely unknown mechanisms. We recently demonstrated that voluntary wheel running showed over 60% reduction in tumor incidence and growth across several murine tumor models. Microarray analysis revealed exercise-induced up-regulation of pathways associated with immune function, prompting further investigations. Immune cell infiltration was significantly increased in tumors from exercising mice, and depletion of NK cells enhanced tumor growth and blunted the exercise-dependent suppression. Moreover, NK cells were engaged through an epinephrine-dependent mobilization, and blockade of this pathway by β-adrenergic blockade blunted the exercise-dependent tumor inhibition. Together these results link exercise with improved immunological control of tumor progression, suggesting that exercise could be a beneficial partner for immunotherapy.

### World-wide immunoscore task force

#### K7 Implementation of the immunoscore in a pathology lab based on the LEAN management system

##### Alessandro Lugli, Heather Dawson, Annika Blank, Inti Zlobec

###### Institute of Pathology, University of Bern, Bern, Switzerland

####### **Correspondence:** Alessandro Lugli - alessandro.lugli@pathology.unibe.ch

*Journal of Translational Medicine* 2016, **15(Suppl 1)**:K7

**Background:** A main goal of a modern Clinical Pathology Division is to provide clinicians involved in the treatment of tumor patients with fast and precise histopathology reports that include information on the TNM stage and potential prognostic and predictive biomarkers. In colorectal cancer, the immunoscore based on immune markers CD3 and CD8 is a promising prognostic approach using immunohistochemistry and a digital scoring system. Nevertheless, there is still the challenge of implementing the immunoscore in daily diagnostic practice in surgical pathology. The aim of the study is to present one of the first histopathology labs designed upon the LEAN management system which will allow a pragmatic and solid implementation of the immunoscore.

**Materials and methods:** Our Clinical Pathology Division was organized into three subunits (MDs, technicians and secretaries) and coordinated strategically and operatively by a staff supported by a LEAN Officer. The following measurements were introduced: reconstruction of the histopathology lab based on the LEAN principles such as standardized working places and continuous workflow, introduction of a diagnostic wing including six sign-out rooms for the MDs and monitoring of the diagnostic process by key performance indicators such as turn-around-times (TAT).

**Results:** The distance travelled by a resection specimen was shortened from 561 to 56 m, batch processing dramatically reduced specimen waiting times and the TAT were reduced from 90 h to an average of 48 for resection specimens and from 55 to 32 h for biopsies, respectively. A LEAN histopathology lab allows a proposal for an Immunoscore Workflow definition: (1) selection of the tumor block by a pathologist; (2) staining by the immunohistochemistry lab; (3) scanning and preparation of the stained slides by technicians; (4) control and reporting by a pathologist.

**Conclusions:** The implementation of the immunoscore into surgical pathology on the operative level needs a well-defined strategy which is simple, applicable and user-friendly. Therefore, it is mandatory that surgical pathology divisions and digital pathology development units test novel and promising digital scoring systems before the implementation in the daily diagnostic practice based on working processes using SOPs and TATs.

### System biology session: Molecular

#### K8 MicroRNAs regulate resistance to target therapy in melanoma

##### Luigi Fattore^1^, Susan Costantini^1^, Mario Acunzo^2^, Giulia Romano^2^, Giovanni Nigita^2^, Alessandro Laganà^3^, Debora Malpicci^4^, Ciro Francesco Ruggiero^4^, Maria Elena Pisanu^1^, Alessia Noto^5^, Claudia De Vitis^5^, Carlo Maria Croce^2^, Paolo Antonio Ascierto^1^, Rita Mancini^5^, Gennaro Ciliberto^1^

###### ^1^Istituto Nazionale per lo Studio e la Cura dei Tumori “Fondazione G. Pascale”, Naples, Italy, ^2^Department of Molecular Virology, Immunology and Medical Genetics, The Ohio State University Comprehensive Cancer Center, Columbus, OH, USA, ^3^Department of Genetics and Genomic Sciences Icahn School of Medicine at Mount Sinai New York, NY, USA, ^4^Dipartimento di Medicina Sperimentale e Clinica, Università degli Studi di Catanzaro “Magna Graecia”, Catanzaro, Italy, ^5^Dipartimento di Medicina Clinica e Molecolare, Sapienza Università di Roma, Rome, Italy

####### **Correspondence:** Gennaro Ciliberto - g.ciliberto@istitutotumori.na.it

*Journal of Translational Medicine* 2016, **15(Suppl 1)**:K8

Although melanoma patients with BRAF mutations benefit from the therapy with BRAF/MEK inhibitors, they invariably develop resistance. Our laboratory is interested in the study of the mechanisms responsible for the establishment of drug resistance, and in particular in non-mutational adaptive changes. Since miRNAs are powerful post-transcriptional regulators of gene expression and play a key role in cancer we decided to assess their possible involvement. Using a bioinformatic approach we recently identified miR-579-3p, as a potential regulator of melanoma progression. Low miR-579-3p expression correlates with disease progression and expression is further decreased in BRAFi/MEKi resistant melanoma cells. miR-579-3p suppresses melanoma cell growth and migration while enhancing apoptosis alone or in combination with BRAF and/or MEKi. Moreover, miR-579-3p forced expression impairs the establishment of resistance to BRAFi. Mechanistically miR-579-3p acts by targeting both BRAF, and MDM2 oncogenes. In matched tumor samples from melanoma patients before and after development of resistance, we observed a reciprocal regulation of the expression of miR-579-3p and of BRAF and MDM-2. More recently using the Nanostring™ platform we started to assess changes in the whole miRNome profile during the development of drug resistance in vitro in two different BRAF-mutated melanoma cell lines. Bioinformatic analysis of Nanostring™ data revealed a stepwise deregulation of a growing number of miRNAs. Pathway analysis uncovered rewiring of a complex network of intracellular pathways capable to affect the expression of secreted pro-inflammatory and pro-angiogenic cytokines, which was confirmed empirically. In summary our findings identify a network of deregulated miRNAs as major players in the establishment of drug resistance in melanoma.

### Combination strategy session

#### K9 Where are we really with clinical trials of combination immunotherapy?

##### Michael Postow

###### Memorial Sloan Kettering Cancer Center, Rockville Centre, NY, USA

####### **Correspondence:** Michael Postow - PostowM@mskcc.org

*Journal of Translational Medicine* 2016, **15(Suppl 1)**:K9

An incredible number of clinical trials are testing various immunotherapy agents in combination strategies. This talk will summarize where we stand with various immunotherapy combinations and stress general principles to consider in ongoing and future combination immunotherapy studies. Although this talk will include data for the ipilimumab and nivolumab combination, many other combination immunotherapy regimens will be discussed.

### System biology session: Immunology

#### K10 Bioinformatic approaches to investigate mechanisms driving the non-T cell-inflamed tumor microenvironment

##### Jason Luke

###### University of Chicago Medicine, Illinois, USA

####### **Correspondence:** Jason Luke - jluke@medicine.bsd.uchicago.edu

*Journal of Translational Medicine* 2016, **15(Suppl 1)**:K10

A subset of patients with cancer have evidence of a spontaneous anti-tumor immune response and T cell infiltration into tumor sites. This can be quantified most robustly via gene expression profiling and has been described as the “T cell-inflamed tumor microenvironment”. This phenotype has prognostic significance and is associated with the presence of microenvironmental factors such as PD-L1, IDO and T regulatory cells. Clinical response to immunotherapeutics such as anti-CTLA4 and anti-PD1 antibodies in multiple cancers is highly associated with the T cell-inflamed tumor microenvironment. Alternatively, the non-T cell-inflamed microenvironment is unresponsive to current immunotherapy approaches. With the development of multi-dimensional genomic databases such as The Cancer Genome Atlas, the potential has arisen to use large scale bioinformatic approaches to investigate differences between tumors on a molecular level. Using a 160 gene T cell based RNA signature as a phenotype, strong associations between tumor-intrinsic signaling pathways, germline single-nucleotide polymorphisms and commensal microbiome have been identified with the presence or absence of the T cell-inflamed tumor microenvironment. These findings are being validated in vivo. Through this frame work rationale combination immunotherapy drug development directed toward either the T cell-inflamed or non-T cell-inflamed tumor microenvironment is being advanced in clinical trials. Specific focus is given to the next major hurdle in cancer immunotherapy: overcoming the non-T cell-inflamed tumor microenvironment.

#### K11 Advances in dendritic cell cancer immunotherapy

##### David Stroncek, Luciano Castiello, Wenjing Chen, Ping Jin, Jiaqiang Ren and Marianna Sabatino

###### Cell Processing Section, Department of Transfusion Medicine, Clinical Center, NIH Bethesda, Maryland, USA

####### **Correspondence:** David Stroncek - dstroncek@cc.nih.gov

*Journal of Translational Medicine* 2016, **15(Suppl 1)**:K11

**Background:** Dendritic cell (DC) vaccines are in clinical trials for several types of cancer. Our laboratory has been working to improve DC vaccine therapy through the discovery of potency markers and improving vaccine production methods.

**Materials and methods:** We have been treating patients with prostate cancer using DCs pulsed with T cell receptor γ alternate reading frame protein (TARP) peptides and patients with breast and other human epidermal growth factor receptor 2 (HER2/*neu*) expressing cancers with DCs transduced with an adenoviral vector expressing HER2/*neu* (adHER2/*neu*). For both clinical trials DCs are being manufactured using peripheral blood mononuclear cell (PBMC) concentrates that have been enriched for monocytes using counter-flow elutriation. The monocytes are cultured with IL-4 and GM-CSF to produce immature DCs and then with lipopolysaccharide (LPS) and interferon gamma to produce mature DCs. During the 4 days required to manufacture the DCs the cells are cultured in RPMI-1640 media supplemented with 10% autologous plasma. We evaluated the transcriptome, secretome and phenotype of TARP DCs and investigated DC characteristics associated with clinical and immunological responses. DC showing a lower expression of a tolerogenic gene signature induced a strong antigen-specific immune response as well as slowing in PSA velocity, a surrogate for clinical response. DCs from patients with clinical and immunological responses were also characterized by lower surface expression of CD14, lower secretion of IL-10 and MCP-1 (CCL2) and a greater secretion of MDC (CCL22).

**Results:** During the manufacture of adHER2/*neu* DCs we found for some patients that the use of autologous plasma as a culture media protein supplement was associated with poor levels of HER2/*neu* expression. The production of DCs with monocytes from these same patients using plasma from healthy subjects yielded high levels of gene expression. A mixture of healthy donor and patient plasma resulted in low transduction levels indicating a factor in patient plasma inhibited gene transfer. Inhibitory factors were found in the plasma of 8 of 21 patients treated. A comparison of adHER2/*neu* DCs manufactured with inhibitory patient and healthy donor plasma found that DCs manufactured with inhibitory plasma had greater expression of CCR7 (CD197) and CD14 and were enriched in IL-10 and IL-6 pathways genes. These results suggest that cytokines in the serum of cancer patients effects that production of DC vaccines and these factors may contribute to systemic immune suppression.

**Conclusion:** In conclusion, the expression CD14 and the secretion of IL-10, CCL2 and CCL22 may be useful DC vaccine potency markers. While the use of autologous plasma for DC manufacturing prevents the exposure of the vaccine recipient to transfusion transmitted diseases, autologous plasma from some patients contains factors that affect adenoviral vector gene transfer and possibly the function of DCs. Further study of these inhibitor factors is warranted to determine if they may contribute to cancer-induced systemic immune modulation.

### Biomarkers session

#### K12 HLA class I antigen processing machinery defects in malignant cells and response to immunotherapy with checkpoint inhibitors

##### Soldano Ferrone

###### Department of Surgery, Massachusetts General Hospital, Harvard Medical School, Boston, MA, USA

####### **Correspondence: **Soldano Ferrone - SFERRONE@PARTNERS.ORG

*Journal of Translational Medicine* 2016, **15(Suppl 1)**:K12

It has been known for some time that malignant transformation of cells may be associated with defects in the expression and/or function of HLA class I antigen processing machinery (APM). This machinery plays a crucial role in the synthesis and expression of HLA class I antigen-tumor antigen (TA) derived peptide complexes. These complexes mediate the recognition of tumor cells by cognate T cells. Multiple molecular mechanisms have been shown to underlie defects in the expression and/or function of HLA class I APM. These defects may cause abnormalities in the synthesis and/or expression of HLA class I antigen-TA derived peptide complexes. These abnormalities may lead to a defective or lack of recognition of tumor cells by cognate T cells, thus providing tumor cells with an escape mechanism from host’s immune system. These functional abnormalities have been suggested to represent a mechanism underlying the association found in many types of malignancies between down regulation or lack of HLA class I APM component(s) in tumor cells and poor clinical course of the disease.

This presentation will discuss (1) the steps which may lead to the generation of tumors with defects in HLA class I APM component expression and/or function with special emphasis on the role of selective pressure in the generation of tumors with this phenotype and (2) the potential role of HLA class I APM defects in the innate and acquired resistance to immunotherapy with checkpoint inhibitor-specific monoclonal antibodies. In addition strategies to overcome this resistance will be described.

## Melanoma Bridge 2016: oral presentations

### Evolving topics in cancer immunotherapy

#### O3 Altering the gut microbiota to improve responses to immune checkpoint blockade

##### Connie P. M. Duong^1,2^, Marie Vetizou^1,2^, Laurence Zitvogel^1,2,3,4^

###### ^1^Gustave Roussy Cancer Campus, Villejuif, France, ^2^INSERM Unit U1015, Villejuif, France, ^3^Université Paris Sud, Université Paris-Saclay, Faculté de Médecine, Le Kremlin Bicêtre, France, ^4^Center of Clinical Investigations in Biotherapies of Cancer 507, Villejuif, France

####### **Correspondence:** Connie P. M. Duong - CONNIE.DUONG@gustaveroussy.fr

*Journal of Translational Medicine* 2016, **15(Suppl 1)**:O3

**Background:** The targeting of the immune checkpoint CTLA4 in melanoma patients has led to increased objective response rates and subsequent approval by the EMA and FDA. However, studies have also reported that some patients have experienced immune-related adverse events, often occurring at sites exposed to commensal microbiota, leading to cessation of treatment. Our lab and others have demonstrated that the gut microbiota impacts the efficacy of checkpoint blockade therapy [1, 2]. We have shown that *Bacteroides* species play an important role on the efficacy of CTLA4 blockade in the treatment of melanoma. Analysis of feces from metastatic melanoma patients led to the identification of three clusters (A, B and C) based on genus composition. Here we present a follow up study, where we hypothesise that compensating Cluster B patient transplanted mice with live or immunogenic bacteria, termed oncomicrobiotics, can increase the efficacy of anti-CTLA4 treatment. Given recent data showing that the co-blockade of CTLA4 and PD1 has a significantly greater response rate compared to targeting CTLA4 alone [3], we also wanted to investigate the impact of microbiota on this combination.

**Materials and methods:** To test this, SPF mice were treated with or without a cocktail of antibiotics (ATBx) composed of ampicillin, streptomycin and colistin. Mice were subsequently gavaged with patient feces from different clusters and inoculated with the sarcoma cell line, MCA205. Mice were then treated with five doses of anti-CTLA4 Ab, gavaged with defined species of oncomicrobiotics and monitored for tumor growth and survival. In order to evaluate the effect of the gut microbiota on the combination, mice were treated with or without ATBx, inoculated with either MCA205 or the melanoma cell line RET, and treated with anti-CTLA4, anti-PD1, or a combination of both Abs.

**Results:** Consistent with our previous data, mice which received feces from Cluster C patients had a significantly greater response to CTLA4 blockade compared to mice which received Cluster B feces. Mice that has been colonised with Cluster B feces had a significantly greater response to anti CTLA4 therapy when compensated with oncomicrobiotics. We also found that the efficacy of anti CTLA4 and anti PD1 blockade is partially dependent on an intact microbiome, as dysbiotic mice had a significantly reduced response to the combination treatment.

**Conclusions:** It is anticipated that this work will increase the therapeutic coverage of immune checkpoint blockade treatment when appropriate oncomicrobiotics are co-administered, resulting in increased durable responses in patients with metastatic melanoma.

**References**

1. Vetizou et al. Anticancer immunotherapy by CTLA-4 blockade relies on the gut microbiota. Science. 2015;350:1079–84.

2. Sivan et al. Commensal *Bifidobacterium* promotes antitumor immunity and facilitates anti-PD-L1 efficacy. Science. 2015;350:1084–89.

3. Postow et al. Nivolumab and Ipilimumab versus Ipilimumab in Untreated Melanoma. NEJM. 2015;37(21):2006–17.

### System biology session: Molecular

#### O4 Inhibition of Stearoyl-Coa desaturase 1 (SCD1) enzymatic activity reverts BRAFi and MEKi-induced selection of cancer stem cells in BRAF-mutated malignant melanoma

##### Maria Elena Pisanu^1,2^, Alessia Noto^2,3^, Luigi Fattore^1,2^, Debora Malpicci^2,4^, Gennaro Ciliberto^1^, Rita Mancini^2,3^

###### ^1^Istituto Nazionale per lo Studio e la Cura dei Tumori “Fondazione G. Pascale”, Naples, Italy, ^2^Dipartimento di Chirurgia “P. Valdoni”, Sapienza Università di Roma, Rome, Italy, ^3^Dipartimento di Medicina Clinica e Molecolare, Sapienza Università di Roma, Rome, Italy, ^4^Dipartimento di Medicina Sperimentale e Clinica, Università degli Studi di Catanzaro “Magna Graecia”, Catanzaro, Italy

####### **Correspondence:** Maria Elena Pisanu - mariaelena.pisanu@uniroma1.it

*Journal of Translational Medicine* 2016, **15(Suppl 1)**:O4

**Background:** Therapy of melanoma has been improved by the advent of immunological checkpoint inhibitors and by targeted therapies with kinase inhibitors [1]. However, the efficacy of targeted therapies against BRAF^V600^ mutations is hampered by the development of acquired resistance [2–3]. Treatment failures in melanoma patients have been attributed in part to the presence of cancer stem cells (CSCs). Observations that CSC have a distinct biology when compared to that of the bulk tumor cells and, more importantly, are resistant to chemotherapies, suggest their involvement in invasion, metastasis and relapse [4]. We previously demonstrated that lung CSCs are enriched for the expression of SCD1, a key enzyme of lipid metabolism involved in the conversion of saturated into mono-unsaturated fatty acids, and that its inhibition suppresses the ability to form spheroids and selectively kills CSCs[5]. In this study we investigated the involvement of SCD1 in melanoma stem cells survival and the interplay between BRAF/ERK inhibitors (BRAF/MEKi) and lipid metabolism.

**Materials and methods:** Bioinformatics analysis of published melanoma datasets was performed using online tool (http://www.cbioportal.org/). SCD1 gene expression data of 479 patients were downloaded from TCGA and correlated with survival. Combination of BRAF/MEKi and the SCD1 inhibitor MF438 were tested by spheroid forming assays on two BRAF-mutated melanoma cell lines (M14 and WM115) grown in selective 3D medium. SCD1, Nanog, Oct4 expression was studied by WB and RT-PCR.

**Results:** Bioinformatics analysis of public datasets was carried out to assess if SCD1 can act as prognostic factor in melanoma. By using online tool we found that overall survival of patients affected by melanoma was inversely correlated to SCD1 expression. This finding led us to measure SCD1 expression in CSCs obtained from WM115 and M14 cell lines. Preliminary results showed that SCD1 expression is upregulated in spheroids derived from both lines. Moreover SCD1 overexpression was associated with enrichment of stemness markers. Exposure to high doses 10–20 μM of Vemurafenib or MEK162 induced increased spheroid formation. Based on these evidences we analyzed the functional role of SCD1 by inhibiting its activity using MF438. We found that spheroids were resistant to BRAF/MEKi and expressed high levels of Oct4 and Nanog. Furthermore we observed that combining BRAF/MEKi with MF438 inhibited synergistically spheroid growth.

**Conclusions:** Our results suggest that treatment of BRAF-mutated melanoma cells with BRAF/MEKi selects for cells with stem cell features and that this phenomenon is counteracted by SCD1 inhibitors. These findings have potential implication of the development of new combination therapies.

**References**

1. Flaherty KT. BRAF inhibitors and melanoma. Cancer J. 2011;17(6):505–511.

2. Lo RS. Combinatorial therapies to overcome B-RAF inhibitor resistance in melanomas. Pharmacogenomics. 2012;13(2):125–8.

3. Corcoran RB, Settleman J, Engelman JA Potential therapeutic strategies to overcome acquired resistance to BRAF or MEK inhibitors in BRAF mutant cancers. Oncotarget 2011;2(4):336–46.

4. Grasso C, Anaka M, Hofmann O, Sompallae R, Broadley K, Hide W, Berridge MV, Cebon J, Behren A, McConnell MJ Iterative sorting reveals CD133+ and CD133-melanoma cells as phenotypically distinct populations. BMC Cancer 2016;16(1):726.

5. Noto A, Raffa S, De Vitis C, Roscilli G, Malpicci D, Coluccia P, Di Napoli A, Ricci A, Giovagnoli MR, Aurisicchio L, Torrisi MR, Ciliberto G, Mancini R Stearoyl-CoA desaturase-1 is a key factor for lung cancer-initiating cells. Cell Death Dis. 2013;4:e947.

#### O5 Screening of malignant melanoma (MM) by miRNA: preliminary data on incidence after initial clinical evaluation

##### Marcella Occelli^1^, Carolina Cauchi^1^, Grazia Sciancalepore^2^, Cristiana Lo Nigro^1^, Michela Rovera^3^, Chiara Varamo^1^, Daniela Vivenza^1^, Zelda Seia^4^, Stefania Palazzini^4^, Fabiana Errico^4^, Davide Basso^4^, Laura Quaranta^4^, Giuseppe Forte^2^, Fulvio Lavagna^5^, Silvia Violante^1^, Paolo Bosio^6^, Laura Lattanzio^1^, Marco Carlo Merlano^1^

###### ^1^Oncologia Medica, Azienda Ospedaliera Santa Croce e Carle, Cuneo, Italy, ^2^Anatomia Patologica, Azienda Ospedaliera Santa Croce e Carle, Cuneo, Italy, ^3^CAS, Azienda Ospedaliera Santa Croce e Carle, Cuneo, Italy, ^4^LILT, Cuneo, Italy, ^5^SS Day Surgery, Azienda Ospedaliera Santa Croce e Carle, Cuneo, Italy, ^6^Chirurgia Generale, Azienda Ospedaliera Santa Croce e Carle, Cuneo, Italy

####### **Correspondence:** Marcella Occelli - marcellaoccelli@gmail.com

*Journal of Translational Medicine* 2016, **15(Suppl 1)**:O5

**Background:** The incidence of MM progressively increases and today 16 cases/100,000 cases per year are expected in Italy. However, there are major differences between geographical areas. In northern Italy the risk is double of that in the southern regions. The screening campaigns led to the identification of many early forms of MM, resulting in increased incidence of the disease, but have not changed mortality. This is possibly related to the identification of melanomas that would have remained silent. It should be remembered that the visit and the dermoscopy are operator-dependent methods and that requires long experience for their optimal use. Even in the most experienced hands, the sensitivity of these methods does not exceed 85% and 90%, respectively. The identification of new effective screening methods, able to eliminate the existing limits, is needed. MM is the skin cancer with the worst prognosis, especially when discovered at advanced stages. Moreover, it would be clinically relevant to identify in advance the melanoma lesions at risk of recurrence.

**Materials and methods:** In 2014 we have activated an ongoing experimental study to investigate the possible role of miRNA in MM screening tool. All the subjects clinically screened (including dermatoscopy) by the “Lega Italiana per la Lotta contro i Tumori” (LILT) are invited to join the study. They undergo a blood sample collection and a panel of 15 miRNAs is analyzed. The primary objective is to compare the level of miRNAs in patients with MM and non-neoplastic skin pigmented lesions, to evaluate their diagnostic value. The study foresees to enroll 700 subjects, to find at least 100 MM.

**Results:** From September 2014 to April 15, 2016, we accrued 546 subjects, who had a suspicious skin lesion. 5 people have not performed excision (patient’s refusal or dermatological second opinion); 87 (16%) had a histological diagnosis of MM. Specifically we found 37 in situ MM, 50 infiltrating, including 15 pT1a, 19 pT1b, 4 pT2a, 2 pT2b, 1 pT3a, 4 pT3b, 2 pT4b. One patient was diagnosed with multifocal mucosal melanoma; in 2 MM patients pathological staging is under progress.

**Conclusions:** The study is ongoing and biomolecular data are not yet available. However, it gives in a prospectic way, preliminary information about the incidence of MM in a population clinically screened and carrying suspected skin lesions.

**Acknowledgements:** This project is supported by CRC Foundation and LILT.

### System biology session: Immunology

#### O6 Regulation of T cell sensitivity by TCR-proximal signaling components during anti-melanoma responses

##### Duane Moogk^1^, Shi Zhong^1,2^, Zhiya Yu^3^, Ivan Liadi^4^, William Rittase^5^, Victoria Fang^1,6^, Janna Dougherty^1^, Arianne Perez-Garcia^1,7^, Iman Osman^1,8^, Cheng Zhu^5^, Navin Varadarajan^4^, Nicholas P. Restifo^3^, Alan Frey^9^, Michelle Krogsgaard^1,10^

###### ^1^Laura and Isaac Perlmutter Cancer Center New York University School of Medicine, New York, NY, 10016, USA, ^2^Life Sciences Center, Xiangxue Pharmaceutical Co., Ltd. GuangZhou, China, ^3^Center for Cancer Research, National Cancer Institute, US National Institutes of Health, Bethesda, MD, 20892, USA, ^4^Department of Chemical and Biomolecular Engineering, University of Houston, TX, 77004, USA, ^5^George W. Woodruff School of Mechanical Engineering, Georgia Institute of Technology, Atlanta, Georgia, 30332-0405, USA, ^6^NYU Medical Scientist Training Program, New York, NY, 10016, USA, ^7^Kite Pharma, Santa Monica, CA, 90404, USA, ^8^Ronald Perelman Department of Dermatology, NYU School of Medicine, New York, NY, 10016, USA, ^9^Department of Cell Biology, New York University School of Medicine, New York, NY, 10016, USA, ^10^Department of Pathology, New York University School of Medicine, New York, NY, 10016, USA

####### **Correspondence:** Michelle Krogsgaard - Michelle.Krogsgaard@nyumc.org

*Journal of Translational Medicine* 2016, **15(Suppl 1)**:O6

**Background:** Immunotherapies for cancers, including melanoma, have made great strides in recent years, yet new and improved approaches are required to achieve more durable responses in a greater number of patients. The in vitro expansion phase of adoptive T cell therapy prior to reinfusion into the patient provides the opportunity to genetically enhance T cell subsets to improve in vivo performance. While the most common genetic modification is the incorporation of engineered antigen-specific TCRs or chimeric antigen receptors [1, 2], modification to signaling pathways in T-cell memory subsets in order to enhance T cell sensitivity is an underexplored strategy. This is mainly because contributions of TCR signaling components that confer differences in activation sensitivity and functional outcomes between CD8+ T_cm_ and T_em_ are unclear.

**Materials and methods:** To understand how TCR-proximal signaling differs significantly between T-cell memory subsets, we derived T_CM_ and T_EM_ [3] from the humanized TCR-transgenic melanoma mouse model (JR209) [4]. We quantified differences in TCR activation and feedback regulation by novel live-cell imaging technologies, phospho-specific protein assays and used modeling of early TCR signaling to reveal the physiological significance of these differences [5].

**Results:** One of the critical steps of T cell triggering is the coordinated phosphorylation and binding of CD3 and Zap-70 by Lck following TCR ligation by Pmhc [6, 7]. Here, we show that T_cm_ and T_em_ possess differential constitutive Lck activities [8]. Immediately proximal to Lck signaling, we observed enhanced Zap-70 phosphorylation in TEM following TCR ligation compared with TCM. Further, we observed increased intracellular calcium influx and cytotoxic effector function in TEM compared with TCM, and provide evidence that this results from a lower probability of TCM reaching threshold activation signaling due to the decreased magnitude of TCR-proximal signaling. We show that the differences in Lck constitutive activity between CD8+ T_cm_ and T_em_ are driven in part by differential regulation by SH2 domain-containing phosphatase-1 (Shp-1) and C-terminal Src kinase (Csk). We demonstrate that inhibition of Shp-1 results in increased constitutive Lck and cytotoxic activity in T_CM_ to levels similar to that of T_EM_.

**Conclusions:** Together, this work demonstrates that differential activities of TCR-proximal signaling components may contribute to establishing the divergent effector properties of T_CM_ and T_EM_. Inhibition of negative regulatory molecules, for example Shp-1 or Csk, or generalized augmentation of T cell sensitivity with miRNA offer potential therapeutic approaches in T cell immunotherapy but must be considered in the context of specificity and optimal targeting.

**References**

1. Kershaw MH et al. Clinical application of genetically modified T cells in cancer therapy. Clin Transl Immunology. 2014;3(5):e16.

2. Hinrichs CS, Rosenberg SA. Exploiting the curative potential of adoptive T-cell therapy for cancer. Immunol Rev. 2014;257(1):56–71.

3. Klebanoff CA et al. IL-15 enhances the in vivo antitumor activity of tumor-reactive CD8 + T cells. Proc Natl Acad Sci USA. 2004;101(7):1969–74.

4. Yu Z et al. Poor immunogenicity of a self/tumor antigen derives from peptide-MHC-I instability and is independent of tolerance. J Clin Invest. 2004;114(4):551–60.

5. Moogk D et al. Constitutive Lck activity drives sensitivity differences between CD8+ memory T cell subsets. J Immunol. 2016;197(2):644–54.

6. Straus DB, Weiss A. Genetic evidence for the involvement of the lck tyrosine kinase in signal transduction through the T cell antigen receptor. Cell. 1992;70(4):585–93.

7. Iwashima M et al. Sequential interactions of the TCR with two distinct cytoplasmic tyrosine kinases. Science. 1994;263(5150):1136–9.

8. Nika K et al. Constitutively active Lck kinase in T cells drives antigen receptor signal transduction. Immunity. 2010;32(6):766–77.

### Biomarkers session

#### O7 Tumor-infiltrating immune cells as potential predictive markers of response to treatment and survival in metastatic melanoma patients receiving ipilimumab

##### Timea Balatoni^1^, Anita Moho^2^, Timea Sebestyén^3^, Anita Varga^4^, Judit Oláh^4^, Zsuzsanna Lengyel^5^, Gabriella Emri^6^, Gabriella Liszkay^1^, Andrea Ladányi^7^

###### ^1^Department of Oncodermatology, National Institute of Oncology, Budapest, Hungary, ^2^1st Institute of Pathology and Experimental Cancer Research, Semmelweis University, Budapest, Hungary, ^3^Department of Pathology, St. John’s Hospital, Budapest, Hungary, ^4^Department of Dermatology and Allergology, Albert Szent-Györgyi Medical Center, University of Szeged, Szeged, Hungary, ^5^Department of Dermatology, Venerology and Oncodermatology, University of Pécs, Pécs, Hungary, ^6^Department of Dermatology, University of Debrecen, Debrecen, Hungary, ^7^Department of Surgical and Molecular Pathology, National Institute of Oncology, Budapest, Hungary

####### **Correspondence:** Andrea Ladányi - ladanyi@oncol.hu

*Journal of Translational Medicine* 2016, **15(Suppl 1)**:O7

**Background:** Immunotherapeutic modalities of cancer treatment have been increasingly gaining ground in the past few years. Antibodies targeting immune regulatory pathways showed unprecedented clinical efficacy in patients with advanced cancers. Nevertheless, generally only a smaller proportion of patients benefit from these therapies, prompting a search for biomarkers that could help identify patients likely to benefit from the treatment. Although several candidates have been suggested, no validated predictive biomarkers are available yet.

**Materials and methods:** Our study was performed on archived paraffin blocks of surgical samples excised within one year before ipilimumab therapy from patients with metastatic melanoma (30 patients, 1–25 lesions per patient). Eighty-six samples were analyzed, 52 nodal and 34 subcutaneous/cutaneous metastases. Immunohistochemical analysis was performed for detection of 10 immune cell markers (CD8, CD45RO, CD20, CD134, CD137, FOXP3, PD-1, CD16, CD68 and NKp46). Intratumoral density of the labeled cells was determined and evaluated in relation to response to ipilimumab treatment and disease outcome.

**Results:** For most of the markers studied, median immune cell densities were at least 2 times higher in lymph node metastases compared to subcutaneous/cutaneous ones, therefore, the prognostic and predictive associations of immune cell infiltration were evaluated separately in the two groups of metastases as well as in all samples together. Thirteen patients were considered responders showing complete or partial response, or stable disease for at least 6 months. Higher prevalence of immune cells expressing the FOXP3, CD8, CD20, NKp46, or CD134 marker was seen in lymph node metastases of the responders compared to non-responders. Kaplan–Meier analysis of survival revealed that high mean density of immune cells in nodal metastases was associated with significantly longer progression-free and overall survival in the case of the majority of cell types studied. In subcutaneous/cutaneous metastases, on the other hand, correlation with treatment response or with survival was limited to the CD16, CD68 and NKp46 markers.

**Conclusions:** Our findings corroborate previous results indicating stronger response to ipilimumab treatment in cases of an immunologically active tumor microenvironment. Studies on larger patient cohorts are required to prospectively validate infiltrating immune cell densities as biomarkers of ipilimumab efficacy and, possibly, to investigate their predictive value in the case of anti-PD-1/PD-L1 agents as well as of other immunotherapy approaches.

The work was supported by the National Research, Development and Innovation Office NKFI Grant 105132.

#### O8 Identification of five circulating microRNAs with high diagnostic values in cutaneous melanoma

##### Beatrice Polini^1^, Stefano Fogli^1^, Sara Carpi^1^, Barbara Pardini^2^, Alessio Naccarati^2^, Nevio Dubbini^3^, Maria Cristina Breschi^1^, Antonella Romanini^3^, Paola Nieri^1^

###### ^1^Department of Pharmacy, University of Pisa, Pisa, Italy, ^2^Human genetic foundation (HUGEF), Torino, Italy, ^3^Department of Medical Oncology, University Hospital of Pisa, Italy

####### **Correspondence:** Beatrice Polini - beatrice.polini@farm.unipi.it

*Journal of Translational Medicine* 2016, **15(Suppl 1)**:O8

**Background:** Melanoma is the fourth and sixth most common malignancy in men and women, respectively, and the 5-year survival rate critically depends on disease stage (American Cancer Society, 2016). MicroRNAs (miRNAs) are small, non-coding, single-stranded RNAs endogenously produced by the cells, which regulate the expression of hundreds of target genes. Circulating miRNAs actively secreted by tumor cells or released as the consequence of tumor cell death, have been proposed as potential biomarkers in cancer patients because of their stability in body fluids, resistance to endogenous RNase and constant expression in healthy individuals (Mitchell et al. 2008; Chen et al. 2008).

**Materials and methods:** In the current study, the expression profiles of a selected panel of circulating miRNAs were analysed in plasma samples derived from patients and healthy donors to identify possible candidate biomarkers for diagnosis, prognosis and/or surveillance of human cutaneous melanoma. The study protocol was approved by local Ethics Committee and conducted in accordance to the principles of the Declaration of Helsinki. Blood samples were collected from melanoma patients (n = 30) at different disease stages, and from healthy age- and sex-matched volunteers (n = 32). Plasma miRNAs were isolated by miRNeasy Serum/Plasma Kit (Qiagen) and real-time PCR were carried out using miRCURY LNATM Universal RT microRNA PCR system (Exiqon, Denmark). Data analysis was carried out using two different strategies for normalization: Global Mean Normalization (GMN) and NormFinder model.

**Results:** The GMN approach and NormFinder algorithm provided 13 and 7 significantly dysregulated miRNAs (p < 0.05), respectively, and those that resulted significantly dysregulated after normalizations and the Bonferroni correction were selected. Circulating miR-15b-5p (p = 1.34e−05), miR-149-3p (p = 3.40e−12), and miR-150-5p (p = 2.85e−12) were up-regulated, while miR-193a-3p (p = 1.30e−06) and miR-524-5p (p = 2.41e−05) were down-regulated in patients (regardless of disease stage) compared to healthy controls. Linear regression and following receiving operator curves (ROC) analyses were performed to evaluate the diagnostic value of these five selected miRNAs (i.e., the ability to discriminate between cases and controls). The area under ROC curve (AUCs) for individual miRNAs ranged from 0.801 to 0.951. Although the predictive power of all selected miRNAs was clearly demonstrated, miR-150-5p and miR-149-3p gave the best performance (AUCs of 0.9489, 95% CI from 0.8852 to 1.017 and 0.9510, 95% CI from 0.8852 to 1.017, respectively). Noteworthy, predictive performance was further improved when considering the double combination of miR-150-5p and miR-149-3p. The double classifier has indeed an increased area under ROC curve (AUC) of 0.966 (95% CI 0.938–0.994) with 90% sensitivity, 68% specificity.

**Conclusions:** In conclusion, findings of the current pilot study identified five circulating miRNAs in melanoma patients, of which three detected for the first time as circulating in this type of cancer, as potential diagnostic biomarkers with high sensitivity and specificity. In particular, the combined miR-149-3p, miR-150-3p and miR-193a-3p signature showed a high capacity to discriminate between healthy subjects and affected individuals. This feature make the signature suitable to be used in controversial melanoma diagnosis. Indeed, these preliminary data that identify a new panel of three miRNAs, including two identified for the first time as circulating in melanoma, deserve to be validated in a larger number of patients, in order to confirm their diagnostic power not only as a possible support to controversial pathology reports histological results but also in early diagnosis of melanoma.

**References**

1. Luke JJ, Ott PA. Drug, healthcare and patient safety. 2014;6:77–88.

2. Aqeilan RI, Calin G, Croce CM. miR-15a and miR-16-1 in cancer: discovery, function and future perspectives. Cell Death Differ. 2010;17:215–20.

3. Lynam-Lennon N, Maher SG, Reynolds JV. The roles of microRNA in cancer and apoptosis. Biol Rev Camb Philos Soc. 2009;84:55–71

4. Mitchell PS, Parkin RK, Kroh EM et al. Circulating microRNAs as stable blood-based markers for cancer detection. Proc Natl Acad Sci. 2008;105:10513–18.

5. Chen X, Ba Y, Ma L, Cai X, Yin Y, Wang K, et al. Characterization of microRNAs in serum: a novel class of biomarkers for diagnosis of cancer and other diseases. Cell Res. 2008;18: 997–1006.

6. Segura MF, Belitskaya-Levy I, Rose AE, et al. (2010) Clin Cancer Res 16: 1577–86

#### O9 Impact of phosphoinositide 3 kinase and vitamin D3 nuclear receptor single nucleotide polymorphisms on the outcome of malignant melanoma patients

##### Francesca Morgese^1^, Davide Soldato^1^, Silvia Pagliaretta^1^, Riccardo Giampieri^1^, Donatella Brancorsini^2^, Silvia Rinaldi^1^, Mariangela Torniai^1^, Anna Campanati^3^, Giulia Ganzetti^3^, Annamaria Offidani^3^, Alfredo Giacchetti^4^, Giuseppe Ricotti^4^, Agnese Savini^1^, Azzurra Onofri^1^, Francesca Bianchi^1^, Rossana Berardi^1^

###### ^1^Medical Oncology, Università Politecnica delle Marche, Azienda Ospedaliero-Universitaria Ospedali Riuniti Umberto I-GM Lancisi-G Salesi, Ancona, Italy, ^2^Section of Pathological Anatomy and Histopathology, Deparment of Neuroscience, Università Politecnica delle Marche, Azienda Ospedaliero-Universitaria Ospedali Riuniti Umberto I-GM Lancisi-G Salesi, Ancona, Italy, ^3^Dermatology Clinic, Università Politecnica delle Marche, Azienda Ospedaliero-Universitaria Ospedali Riuniti Umberto I-GM Lancisi-G Salesi, Ancona, Italy, ^4^U.O. Dermatologia, INRCA/IRCCS, Ancona, Italy

####### **Correspondence:** Francesca Morgese - francescamorgese85@gmail.com

*Journal of Translational Medicine* 2016, **15(Suppl 1)**:O9

**Background:** Nowadays, the biomolecular mechanisms at the basis of malignant melanoma development and progression are still poorly understood. Although several studies were conducted in order to associate single nucleotide polymorphisms (SNPs) frequencies with the carcinogenesis and tumors outcome, malignant melanoma literature data are inconclusive [1-14].

In our study we evaluate the impact of different genotypes for phosphoinositide 3 kinase (PI3K) and vitamin D3 nuclear receptor (VDR) SNPs on melanoma patients’ outcome.

**Materials and methods:** Genomic DNA of 88 patients was extracted from blood and Formaldehyde Fixed-Paraffin Embedded samples. Gene polymorphisms were determined by Real-Time Polymerase chain reaction (PCR) using TaqMan assays [15]. We selected polymorphisms of the regulatory and catalytic subunit of PI3K encoded by PIK3CA [16] and PIK3R1 [17] genes, respectively. In particular, we analyzed rs2699887C > T of *PIK3CA* and rs3730089G > A of *PIK3R1* SNPs. Furthermore we considered the following *VDR* SNPs: rs2228570A > G (Fok1), rs731236A > G (Taq1) and rs1544410C > T (Bsm1).

Progression free survival (PFS) and overall survival (OS) were estimated with the Kaplan–Meier method and with Mantel–Haenszel log-rank test [18].

**Results:** The statistical analysis for rs2699887C > T of *PIK3CA*, rs3730089G > A of *PIK3R1*, Taq1 and Bsm1 of *VDR* didn’t result in statistical significant differences in PFS and OS.

On the contrary, Fok1 of *VDR* showed a significant difference in PFS after the first line therapy in the analyzed population (median PFS = 21.2 months of homozygous recessive genotype vs. 3.3 months of homozygous dominant and heterozygous ones, *p* = 0.03). In particular, in homozygous recessive patients for Fok1 SNPs of *VDR* a high rate of histological regression and BRAF mutation were observed. Furthermore, more efficacy of BRAF +/- MEK inhibitors therapies vs. homozygous dominant and heterozygous ones was shown.

**Conclusions:** Few studies, from the published literature, associate malignant melanoma patients’ outcome with *VDR* SNPs [7, 19, 20]. Our study demonstrated a significant correlation between homozygous recessive genotype of Fok1 SNPs of VDR gene and an increased PFS in patients who underwent a first line therapy with BRAF inhibitors.

**References**

1. Shastry BS. SNP alleles in human disease and evolution. Journal of Human Genetics 2002;47(11):0561–6.

2. Chalhoub N, Baker SJ. PTEN and the PI3-kinase pathway in cancer. Annu Rev Pathol. 2009;4:127–50.

3. Jurutka PW, Remus LS, Whitfield GK, Thompson PD, Hsieh JC, Zitzer H, Tavakkoli P, Galligan MA, Dang HT, Haussler CA, Haussler MR. The polymorphic N terminus in human vitamin D receptor isoforms influences transcriptional activity by modulating interaction with transcription factor IIB. Mol Endocrinol. 2000;14(3):401–20.

4. Köstner K, Denzer N, Müller CS, Klein R, Tilgen W, Reichrath J. The relevance of vitamin D receptor (VDR) gene polymorphisms for cancer: a review of the literature. Anticancer Res. 2009;29(9):3511-36.

5. Haussler MR, Jurutka PW, Haussler CA, Hsieh J-C, Thompson PD, Remus LS, Selznick SH, Encinas C, Whitfield GK. VDR-mediated transactivation: interplay between 1,25(OH)2D3, RXR heterodimerization, transcription (co)factors and polymorphic receptor variants. In: R. Norman, R. Bouillon, and M. Thomasset (eds.), Vitamin D. Chemistry, Biology and Clinical Applications of the Steroid Hormone. 1997:210–17.

6. Haussler MR, Whitfield GK, Haussler CA, Hsieh JC, Thompson PD, Selznick SH, Dominguez CE, Jurutka PW. The nuclear vitamin D receptor: biological and molecular regulatory properties revealed. J. Bone Miner. Res.1998;13:325–49.

7. Orlow I, Reiner AS, Thomas NE, Roy P, Kanetsky PA, Luo L, Paine S, Armstrong BK, Kricker A, Marrett LD, Rosso S, Zanetti R, Gruber SB, Anton-Culver H, Gallagher RP, Dwyer T, Busam K, Begg CB, Berwick M; GEM Study Group. Vitamin D receptor polymorphisms and survival in patients with cutaneous melanoma: a population-based study. Carcinogenesis. 2016;37(1):30–8.

8. Evans SR, Houghton AM, Schumaker L, Brenner RV, Buras RR, Davoodi F, Nauta RJ, Shabahang M. Vitamin D receptor and growth inhibition by 1,25-dihydroxyvitamin D3 in human malignant melanoma cell lines. J Surg Res. 1996;61(1):127–33.

9. Shabahang M. Vitamin D receptor and growth inhibition by 1,25-dihydroxyvitamin D3 in human malignant melanoma cell lines. J Surg Res. 1996;61(1):127–33.

10. Mason RS, Pryke AM, Ranson M, Thomas HE, Posen S. Human melanoma cells: functional modulation by calciotropic hormones. J Invest Dermatol. 1988;90(6):834–40.

11. Osborne JE, Hutchinson PE. Vitamin D and systemic cancer: is this relevant to malignant melanoma? Br J Dermatol. 2002;147(2):197–213.

12. Gandini S, Raimondi S, Gnagnarella P, Doré JF, Maisonneuve P, Testori A. Vitamin D and skin cancer: a meta-analysis. Eur J Cancer. 2009;45(4):634–41.

13. Zhao XZ, Yang BH, Yu GH, Liu SZ, Yuan ZY. Polymorphisms in the vitamin D receptor (VDR) genes and skin cancer risk in European population: a meta-analysis. Arch Dermatol Res. 2014;306(6):545–53.

14. Hou W, Wan X, Fan J. Variants Fok1 and Bsm1 on VDR are associated with the melanoma risk: evidence from the published epidemiological studies. BMC Genet. 2015;16:14.

15. http://www.ncbi.nlm.nih.gov/snp

16. Bader AG, Kang S, Zhao L, Vogt PK. Oncogenic PI3 K deregulates transcription and translation Nature Reviews Cancer. 2005;5:921–929.

17. Kang S, Bader AG, Vogt PK. Phosphatidylinositol 3-kinase mutations identified in human cancer are oncogenic. PNAS 2005;102(3):802–07.

18. MedCalc Software bvba, Ostend, Belgium, http://medcalc.org.

19. Schäfer A, Emmert S, Kruppa J, Schubert S, Tzvetkov M, Mössner R, Reich K, Berking C, Volkenandt M, Pföhler C, Schön MP, Vogt T, König IR, Reichrath J. No association of vitamin D metabolism-related polymorphisms and melanoma risk as well as melanoma prognosis: a case–control study. Arch Dermatol Res. 2012;304(5):353–61.

20. Newton-Bishop JA, Beswick S, Randerson-Moor J, Chang YM, Affleck P, Elliott F, Chan M, Leake S, Karpavicius B, Haynes S, Kukalizch K, Whitaker L, Jackson S, Gerry E, Nolan C, Bertram C, Marsden J, Elder DE, Barrett JH, Bishop DT. Serum 25-hydroxyvitamin D3 levels are associated with breslow thickness at presentation and survival from melanoma. J Clin Oncol. 2009;27(32):5439–44.

## Melanoma Bridge 2016: poster presentations

### P1 A “special” patient

#### Giovanna Galdo, Gianfranco Orlandino, Salvatore Serio, Domenico Massariello, Tommaso Fabrizio

##### Plastic Surgery Unit, IRCCS-CROB, Rionero in Vulture, PZ, Italy

###### **Correspondence:** Giovanna Galdo - giovanna.galdo@crob.it

*Journal of Translational Medicine* 2016, **15(Suppl 1)**:P1

**Background:** Patients with Down syndrome are at low risk for developing solid tumors—such as carcinoma of the lung, breast and cervix. Testicular cancer would be the only one with a higher risk than the healthy population [1]. We present the case of a patient with Down syndrome with a history of seminoma of the testis, and melanoma of the back.

**Materials and methods:** A male patient, aged 31, suffers from Down’s disease (Trisomy 21 free) with autism, lactose intolerance and with a history of right testicular seminoma (pT2N0) to age of 27 years. Having find this, inguinal orchiectomy was performed followed by an administration of carboplatin with disease control. Four years later he came for one of the follow-up feedback oncological check planned for seminoma, presenting an atypical pigmented lesion of the back during. The nodule had the following dermoscopic features: nodular lesion of hypopigmented area with gray-blue veil, atypical pattern, irregular peripheral blood cells. The patient was subjected to intervention by surgical excision under general anesthesia for suspected melanoma which was followed surgical radicalization and research limphonode sentinel (1.42 mm Breslow, mitotic rate 6 mm^2^).

**Results:** The LNS was negative immunohistochemical investigation and there were no surgical complications (wound dehiscence, sovrinfezione, seroma etc.)

**Conclusion:** Down’s syndrome has an increased risk of onset of solid tumors only in case of seminoma; trisomy 21 would be protective against some solid tumors, such as lung cancer, breast, cervix and melanoma [2]. We report a case of a patient with Down syndrome with a history of testicular seminoma and metachronous occurrence of melanoma on the back.

**Consent to publish:** Authors have written informed consent from the patient to publish.

**References**

1. Hasle H, Friedman JM, Olsen JH, Rasmussen SA. Low risk of solid tumors in persons with Down syndrome. Genet Med. 2016.

2. Forés-Martos J, Cervera-Vidal R, Chirivella E, Ramos-Jarero A, Climent J. A genomic approach to study down syndrome and cancer inverse comorbidity: untangling the chromosome 21. Front Physiol. 2015;6:10.

### P2 Thin and thick primary cutaneous melanomas reveal distinct patterns of somatic copy number alterations

#### Valentina Montagnani^1^, Matteo Benelli^2^, Alessandro Apollo^1^, Chiara Pescucci^2^, Danilo Licastro^3^, Carmelo Urso^4^, Gianni Gerlini^5^, Lorenzo Borgognoni^5^, Lucio Luzzatto^1^, Barbara Stecca^1^

##### ^1^Core Research Laboratory-Istituto Toscano Tumori, Florence, Italy, ^2^Diagnostic Genetics Unit, Careggi University Hospital, Florence, Italy, ^3^CBM, Genomics, Area Science Park, Basovizza, Trieste, Italy, ^4^Anatomic Pathology Unit, Dermatopathology Section, S.M. Annunziata Hospital, Florence, Italy, ^5^Plastic Surgery Unit, S.M. Annunziata Hospital, FL, Italy

###### **Correspondence:** Barbara Stecca - barbara.stecca@ittumori.it

*Journal of Translational Medicine* 2016, **15(Suppl 1)**:P2

**Background:** Melanoma is a highly aggressive form of skin cancer that originates from malignant transformation of melanocytes or from their neural crest-derived multipotent precursors. The aggressive behavior of melanoma is correlated with its histological features, such as the thickness of the primary tumor and the mitotic index. Identification of predisposing genes implicated in melanoma progression is crucial to better understand this disease and improve its treatment. In recent years, genomic sequencing studies have uncovered mutations in multiple genes in melanoma [1-3]. However, the majority of these studies have investigated metastatic or advanced primary melanomas, but data on early stages are very limited. Here we performed a comparative analysis of thin (<1 mm) and thick (>4 mm) melanomas using exome sequencing.

**Materials and methods:** 5 thin (<1 mm) and 5 thick (>4 mm) untreated fresh-frozen primary cutaneous melanomas and their matched peripheral blood were collected and processed for DNA isolation. Exome sequencing was performed using Illumina. Somatic Single Nucleotide Variants (SNVs) and small Insertion/Deletions were analyzed by MuTect, and Somatic Copy Number Alterations (SCNAs) were detected with EXCAVATOR. Two public available microarray data sets (GEO-46517 and GDS3966) were used to assess expression of genes involved in regions of copy loss and gain. The effect of specific mutations or gene copy loss was investigated in vitro using melanoma cell lines.

**Results:** Unsupervised hierarchical clustering analysis of SCNAs identified two groups corresponding to thin and thick melanomas, suggesting distinct genetic differences between those two groups. The most striking difference between them was the much greater abundance of SCNAs in thick melanomas, whereas mutation frequency did not significantly change between the two groups. Interestingly, we found novel mutations and focal SCNAs in genes that are embryonic regulators of axon guidance, predominantly in thick melanomas. Analysis of microarray datasets provided further support for a potential role of Ephrin receptors A4 and A7 in melanoma progression. Among regions with copy loss we identified PARK2, an E3 ubiquitin-protein ligase with putative tumor suppressor role. Functional analysis in melanoma cell lines confirmed that PARK2 silencing enhanced whereas PARK2 over-expression inhibited melanoma cell growth. Finally, we have identified a set of SCNAs, including amplification of *BRAF* and of the epigenetic modifier *EZH2,* that are specific for the group of thick melanomas that developed metastasis during follow-up.

**Conclusions:** Our data reveal that mutations occur early during melanoma development, whereas SCNAs seem to be involved in melanoma progression, and suggest that thin and thick melanomas from the outset might be two different subtypes of melanomas that differ in their tendency to undergo SCNAs.

**References**

1. Mar VJ, Wong SQ, Li J, Scolyer RA, McLean C, Papenfuss AT, Tothill RW, Kakavand H, Mann GJ, Thompson JF, et al. BRAF/NRAS wild-type melanomas have a high mutation load correlating with histologic and molecular signatures of UV damage. Clin Cancer Res. 2013;19:4589–98.

2. Krauthammer M, Kong Y, Bacchiocchi A, Evans P, Pornputtapong N, Wu C, McCusker JP, Ma S, Cheng E, Straub R, et al. Exome sequencing identifies recurrent mutations in NF1 and RASopathy genes in sun-exposed melanomas. Nat Genet. 2015;47:996–1002.

3. Cancer Genome Atlas Research Network et al. Genomic Classification of Cutaneous Melanoma. Cell. 2015;161:1681–96.

### P3 Nivolumab and diabetes mellitus: safe administration in a patient with pancreatic metastases from melanoma

#### Elisabetta Gambale^1^, Camilla Tinari^2^, Alberto Quinzii^1^, Alessio Cortellini^3,4^, Consiglia Carella^5^, Michele De Tursi^1^

##### ^1^Department of Medical, Oral and Biotechnological Sciences, “G. D’Annunzio” University of Chieti-Pescara, Chieti, Italy, ^2^Unit of Endocrinology, Department of Medicine and Sciences of Aging, Ce.S.I.-Me.T., “G.D’ Annunzio” University of Chieti-Pescara, Chieti, Italy, ^3^Medical Oncology, San Salvatore Hospital, L’Aquila, Italy, ^4^Department of Biotechnological and Applied Clinical Sciences, University of L’Aquila, L’Aquila, Italy, ^5^Oncology Unit, “SS. Annunziata” Hospital, Chieti, Italy

###### **Correspondence:** Elisabetta Gambale - gambaleelisabetta@gmail.com

*Journal of Translational Medicine* 2016, **15(Suppl 1)**:P3

**Background:** Anti-programmed cell death-1 (PD-1) antibodies can be a risk factor for insulin dependent diabetes mellitus as a side-effect, probably due to an inappropriate activation of T cells [1, 2, 3]. Very small number of cases have been reported and few data are available about patients treated with anti-PD-1 antibodies with of other risk factors for diabetes, included combined use with other immune modulators, pancreatic metastases and preexisting type 2 diabetes [4].

Here we report our recent experience of a patient underwent pancreatoduodenectomy for pancreatic metastases from melanoma and treated with Nivolumab.

**Case report:** A 45-year-old man was admitted to our Oncology Unit in June 2016 with the diagnosis of metastatic melanoma. ECOG Performance Status was 0 and no comorbidities were reported. In May 2016 pathological lymph nodes in left inguinal region were removed surgically and the histological diagnosis was metastases from melanoma, BRAF mutated. In June 2016, he underwent pancreatoduodenectomy for metastases of melanoma in the head of the pancreas. On July 2016, he started Nivolumab administered at 3 mg/kg intravenously, every 2 weeks. Basal glycemia, islet autoantibodies, haemoglobin A1c and serum C-peptide levels were normal. The last haematological reports, performed on 16 August 2016, showed hyperglycemia (131 mg/dl-reference range:74–106), normal levels of serum C-peptide, absence of islet autoantibodies and elevated haemoglobin A1c level (42 mmol/mol—reference range: 20.0–38.0). At this moment, he continues Nivolumab and he is constantly monitored for his increased risk of diabetes.

**Discussion:** Our research in the literature found only four reports (eight cases) of diabetes mellitus during treatment with anti- PD-1 antibodies and, among these, only a case of patient with pancreatic metastases, probably also due to the rarity of pancreatic metastases from melanoma [3,4,5]. All described reports develop acute severe hyperglycemia with ketoacidosis or low/undetectable C-peptide, with a rapid fall into insulin-dependence; the islet autoantibodies are often negative. Considering the rapid onset, severity and potential mortality of those situations, routine measurement of both haemoglobin A1c and blood glucose levels is necessary during administration of anti-PD-1 antibodies [3]. Our single-patient experience demonstrates that administration of Nivolumab is safe and well tolerated even in presence of risk factors for diabetes. However, further accumulation of cases, evidence and further studies are required to clarify the incidence of diabetes mellitus, the pathogenesis and the background factors and to identify biomarkers predictive of anti-PD-1 therapy-related diabetes, for the prevention and the timely diagnosis of this serious adverse event [3, 4].

**Consent to publish:** Authors have written informed consent from the patient to publish.

**References**

1. Brahmer JR, Tykodi SS, Chow LQ, et al. Safety and activity of anti-PD-L1 antibody in patients with advanced cancer. N Engl J Med 2012;366:2455–65.

2. Robert C, Long GV, Brady B, et al. Nivolumab in previously untreated melanoma without BRAF mutation. N Engl J Med. 2015;372:320–30.

3. Okamoto M et al. Fulminant type 1 diabetes mellitus with anti-programmed cell death-1 therapy. J Diabetes Investig 2016 **(Epub ahead of print)**.

4. Hughes J et al. Precipitation of autoimmune diabetes with anti-PD-1 immunotherapy. Diabetes Care. 2015;38(4).

5. Jana Tet al. Multiple pancreatic metastases from malignant melanoma: Conclusive diagnosis with endoscopic ultrasound-guided fine needle aspiration. Endosc Ultrasound. 2015;4(2):145–8.

### P4 Adverse events associated with “Anti-programmed death-1” therapy for patients with melanoma

#### Adele Emanuela De Francesco^1^, Mariarosanna De Fina^1^, Maria Cristina Zito^1^, Maria Dezia Bisceglia^1^, Stefania Esposito^1^, Giuseppina Fersini^2^

##### ^1^“Mater Domini” University Hospital, Pharmacy Unit, Catanzaro, Italy, ^2^Settore Politiche del Farmaco, Dipartimento Tutela della Salute, Catanzaro, Italy

###### **Correspondence:** Mariarosanna De Fina - mdefina@hotmail.it

*Journal of Translational Medicine* 2016, **15(Suppl 1)**:P4

**Background:** The melanoma is one of the major cancers that occur at a young age. In Italy it is the third most common cause of cancer in people under the age of 50 years old. The incidence of malignant melanoma is on the rise in both men (+3.6%/year) than in women (+3.7%/year).

With the advent of PD-1 inhibitor (anti-programmed-death-1, pembrolizumab, nivolumab) there has been an important evolution in the treatment of advanced unresectable or metastatic melanoma, associated with decrease morbidity and mortality.

Safety and tolerability of the cancer drugs are not well known for the rapid commercialization. This study investigated the occurence and characteristics of adverse drug reactions immunological treatment-related.

**Materials and methods:** A retrospective observational study on suspected adverse drug reactions (ADRs) was conducted from January 2015 to August 2016. We extracted from Pharmacovigilance National Network (RNF) and from the European database of ARDs (Eudravigilance.org) the reactions occurred during melanoma disease treated with pembrolizumab (Keytruda^®^) or nivolumab (Opdivo^®^).

**Results:** In the period of study, 25 ADRs by PD-1 inhibitor are included in the RNF. 7 ADRs reported when PD-1 used as part of malignant melanoma therapy: 6 Keytruda^®^ (4 severe) and 1 Opdivo^®^. The only ADR reported against Opdivo^®^ and considered “ not serious “ highlighted in female patient under 65, resulted in a transient state of thrombocytopenia. 60% of ADRs relating to Keytruda^®^ were found in patients participants in clinical trials. In particular relevant are 2 reports: one severe reactions (diarrhea) and the other non-severe reaction (itching). Both ADRs occurred in patients (M/F 1:1) treatment-experienced with pembrolizumab and in which diagnosed with melanoma by more than 10 years. Although there had been adverse reactions no patient discontinued treatment with Keytruda^®^ or Opdivo^®^. The Opdivo^®^’s ADRs is the only case in both databases. All other ADRs encountered at the national level are in line with those reported in the Eudravigilance.org.

**Conclusion:** Our data showed an acceptable tolerability profile. Every ADR was known and reported in data sheet. Post-marketing surveillance is a research tool needed to deepen the benefit-risk profile, especially in oncology. High toxicity of antineoplastic therapy determines numerous ADRs that are not always reported, in fact in the RNF the number of ADRs reported for this class of drugs is by far lower than that of the other classes.

### P5 The A2B adenosine receptor: an emerging therapeutic target in cancer

#### Silvana Morello, Claudia Sorrentino, Aldo Pinto

##### Department of Pharmacy, University of Salerno, 84084, Fisciano, SA, Italy

###### **Correspondence:** Silvana Morello - smorello@unisa.it

*Journal of Translational Medicine* 2016, **15(Suppl 1)**:P5

**Background:** Adenosine is an ATP metabolite, generated in the extracellular space by the ectonucleotidase CD73. In the tumor microenvironment adenosine impairs anti-tumor immunity, through the Gs-coupled receptors A2A and/or A2B, promoting tumor growth and survival. Blockade of CD73 or A2AR subtype has been shown to improve the anti-tumor immune response. Inhibitors of these targets are currently in Phase I clinical trials in cancer patients. Whilst A2AR is the most thoroughly characterized receptor involved in the immunosuppressive effects of adenosine, A2B is emerging as a potential anti-cancer target.

**Materials and methods:** To investigate the mechanisms through which the A2BR pharmacological modulators impact the primary tumor growth a syngeneic mouse model of melanoma was used. B16.F10 melanoma-bearing mice were treated with a selective antagonist of A2BR, PSB1115. Myeloid and lymphoid leucocyte populations were analyzed in the tumor tissue, tumor draining lymph node and spleen harvested from control and PSB1115-treated groups. The effects of A2BR modulators were also examined in tumor stroma cells, including endothelial cells and melanoma-associated fibroblasts. The therapeutic potential of the A2BR blocker was tested in combination with some chemotherapeutics, such as dacarbazine.

**Results:** Our results show that A2BR inhibition with PSB1115 significantly delayed tumor growth in mice. PSB1115 treatment decreased the accumulation of myeloid-derived suppressor cells (MDSCs) in the tumor microenvironment but not in non-tumoral peripheral organs. This effect was associated with an increased numbers of tumor-infiltrating CD8^+^ T cells and NKT cells, and enhanced levels of IFN-γ and granzyme B. The anti-tumor effects of PSB1115 was indeed lost in melanoma-bearing nude mice lacking of T cells. The effects of A2BR inhibitor was also evaluated on tumor stroma cells. Tumor angiogenesis in PSB1115-treated mice was inhibited and the expression levels of tumor VEGF reduced. Furthermore we found that PSB1115 treatment impacted the function of melanoma-associated fibroblasts in producing FGF2 and CXCL12, which contribute to enhance tumor growth. Treatment of animals with PSB1115 enhanced the anti-tumor activity of dacarbazine and anti-VEGF agents.

**Conclusions:** Pharmacological blockade of A2BR inhibits the growth of syngeneic melanoma in vivo. PSB1115 treatment decreases the number of tumor MDSCs, increases the number of tumor-infiltrating T lymphocytes, affects tumor angiogenesis and the activation of melanoma-associated fibroblasts. Therefore data from animal studies support the therapeutic potential of A2B blockers in melanoma.

### P6 CTLA4-blocking antibodies induced a different entity of severe hypophysitis in patients with advanced melanoma: experience of a multidisciplinary team for a better clinical management

#### Antonella Di Sarno^1^, Antonella Bianco^1^, Carmine D’Aniello^1^, Francesca Andreozzi^1^, Lucia Festina^2^, Vito Vanella^2^, Paolo Antonio Ascierto^2^, Vincenzo Montesarchio^1^

##### ^1^UOC of Oncology A.O.R.N. dei Colli, Monaldi Hospital, Naples, Italy, ^2^IRCCS Pascale, Naples, Italy

###### **Correspondence:** Antonella Di Sarno - antonella.disarno@gmail.com

*Journal of Translational Medicine* 2016, **15(Suppl 1)**:P6

**Background:** CTLA4- blocking antibodies induced- hypophysitis is the most frequent endocrine-adverse events (E-AEs) grade ¾ in patients with advanced melanoma. Unlike sporadic hypophysitis, immune checkpoint inhibitors-induced an atypical hypophysitis, which is more common in men than in women, hyperprolactinemia and diabetes insipidus are extremely rare. Finally, visual field defects are rare because swelling is not large enough to affect the optic chiasma.

**Aims:** Given the difficult in accurately diagnosing this atypical hypophysitis and the poor data supporting establishment of standard systemic therapy options, patients were evaluated within the setting of a multidisciplinary team, endocrinologists with expertise in pituitary disease, oncologists and neuroradiologists.

**Materials and methods:** Twenty-one (13 male; age range 27–73 years) out of 97 patients treated with CTLA4-blocking antibodies for advanced melanoma affected by hypophysitis, were studied. Clinical, hormonal and pituitary MRI findings at diagnosis and during the follow-up requires a multidisciplinary approach in order to ensure a personalized treatment approach.

**Results:** Hypophysitis with hypopituitarism occurred after the third and four CTLA4-blocking antibodies infusion in 19 and 2 out of 21 patients, respectively. All 21 patients had at least one hormonal defects: thyrotroph (n. 15), corticotroph (n. 14) and gonadotroph (n.9). None had hyperprolactinemia, diabetes insipidus and visual field defects. Pituitary MRI showed a mild/moderate enlarged gland in 17 and 4 patients, respectively. All patients had rapid improvement of clinical symptoms during the first and second month after the beginning replacement therapy. None of 14 patients showed recovery of corticotroph axis at the end of follow-up (12 months). Ten out of 15 patients recovered thyrotroph axis at 3 months of follow-up.

**Conclusion:** Early diagnosis and close clinical, biochemical and radiological monitoring are mandatory for correct management of E-AEs. Despite other adverse events, hormonal replacement therapy often permits patients to continue immunotherapy. So, as the use of immunotherapy increases, in order to provide an optimal care a multidisciplinary approach is recommended.

### P7 Human antibody fragments revealed unique sialylation pattern on cancer stem cells in melanomas and were used for chimeric antigenic receptor constructs to genetically engineer immune T cells

#### Beatrix Kotlan^1^, Maria Godeny^2^, Farkas Emil^3^, Laszlo Toth^3^, Szabolcs Horvath^4^, Klara Eles^4^, Timea Balatoni^5^, Akos Savolt^3^, Andras Szollar^3^, Miklós Kasler^5^, Gabriella Liszkay^4^

##### ^1^Molecular Immunology and Toxicology, National Institute of Oncology, Budapest, Hungary, ^2^Department of Radiological Department, National Institute of Oncology, Budapest, Hungary, ^3^Center of Oncosurgery, National Institute of Oncology, Budapest, Hungary, ^4^Center of Surgical and Molecular Tumorpathology, National Institute of Oncology, Budapest, Hungary, ^5^Department of Oncodermatology, National Institute of Oncology, Budapest, Hungary, ^6^Board of Directors, National Institute of Oncology, Budapest, Hungary

###### **Correspondence:** Beatrix Kotlan - kotlanb@netscape.net

*Journal of Translational Medicine* 2016, **15(Suppl 1)**:P7

**Background:** Revealing the very small percentage of highly malignant cancer stem cells (CSC) is a major issue for the development of effective cancer therapeutics. Focusing on the natural humoral immune response, human metastatic melanoma tissue sections were investigated in terms of abnormal glycosilation pattern, as it has major importance in tumorproliferation, cancer metastases, invasiveness, angiogenesis, signal transfers and apoptosis.

**Materials and methods:** Freshly removed surgical specimen (n = 134) were processed for immunohistochemistry, for antibody profile analysis with tumor infiltrating B (TIL-B) cell Phage Display Assay, genexpression studies and/or cell cultures. Results: Our immunohistochemical findings allowed us to develop a new strategy to reveal cancer stem cells by unique tumor-associated disialylated glycosphingolipids (GD3 gangliosides) and their 9-O acetyl derivatives. The novel antibody profile analysis enabled the detection and selection of those human antibody variable region genes that have the specificity to bind to these uniquely disialylated glycosphingolipids strongly expressed on spheroid forming CSCs. Other available cancer stem cell markers (CD133, Nestin, CD34, ABCB5) were defined paralelly. The selected TIL-B cell originated GD3 single chain Fv (scFv) antibody variable gene regions became candidates for new chimeric antigen receptor (CAR) third generation constructs with non viral vector system to transfect cancer targeting immune T cells. Fifteen percent of transfected cells could be seen by immunofluorescence labelling, FACS analysis.

**Conclusion:** A new strategy is shown here to define scFv-s with unique specificities to tumor-associated GD3 gangliosides on cancer stem cells. For designing human GD3 CARs as new cancer stem cell targeting tools, the transposone-based gene delivery system showed advantageous characteristics. These results have strong cancer therapeutic importance due to using scFv of human origin and the cancer stem cell targeting potential.

**Acknowledgements:** Fulbright Grants Nos 1206103 and 1214104, Harry J. Lloyd Charitable Trust Melanoma Research Award, Hungarian OTKA T030380 and the valuable help of Dr. A Korngold, Dr. T. J. Laskowski, Dr. B. Jena, Dr N. Belousova and Prof Dr. L. J. N. Cooper are acknowledged.

### P8 Cutaneous melanoma microenvironment: a two-dimensional topographical computer-aided analysis as a perspective for new therapeutic strategies

#### Daniel Yiu^1^, Fabio Grizzi^2^, Federica Patrinicola^2^, Maurizio Chiriva-Internati^3^, Stefania Motta^4^, Marcello Monti^4^

##### ^1^Humanitas University, Rozzano, Milan, Italy, ^2^Department of Immunology and Inflammation, Humanitas Clinical and Research Hospital, Rozzano, Milan, Italy, ^3^Kiromic LLC, Houston, Texas, USA, ^4^University of Milan, Milan, Italy

###### **Correspondence:** Daniel Yiu - daniel.yiu@hotmail.com

*Journal of Translational Medicine* 2016, **15(Suppl 1)**:P8

**Background:** One of the concepts raised and defined by the “Immunotherapy Summit” [1] as critical was the need to identify hurdles that impede effective translation of cancer immunotherapy. Among these, the complexity of cancer, tumor heterogeneity and immune escape and the lack of definitive biomarkers for assessment of clinical efficacy [1,2]. Repeated failures in melanoma therapy has made clear that the molecular mechanisms leading to melanoma are still poorly understood and that the main prognostic factors for primary melanoma are Breslow thickness, ulceration and mitotic index [3]. However it is now accepted that the microenvironment surrounding and interacting with the tumoral cell is pivotal in determining the dynamics, organization of cutaneous melanoma and its response to therapy [4]. Here we first apply a combined approach based on a standardized histochemical and immunohistochemical methodology and a computer-aided image analysis systems to investigate how the expression of two Tumor-Associated Antigens (TAAs), i.e. Sperm protein 17 (Sp17) and Squamous Cell Carcinoma Antigen 1 (SCCA1), is related to morphological and physical properties of the surrounding tumor microenvironment in melanoma tissues.

**Materials and methods:** 30 primary melanomas were considered in the present study [Age: 58 ± 15 years (Range: 36-88 years); Breslow index: 4 ± 5 mm, (range: 0.26–22 mm)]. Consecutive paraffin embedded sections were stained by a Sirius red solution and treated with antibodies raised against Sp17, SCCA1 (Proteintech Group, Inc), CD34 and Ki67 (Dako, Italy). All stained slides were analyzed by a computer-aided image system that automatically selects and quantitatively evaluates the immunoreactive entities, their density, distribution, morphology, and distances. Additionally, collagen fibers have been typed as Type I or III, using a polarized light microscopy, and their alignment determined.

**Results:** We first evidenced that the expression of SP17 and SCCA1 in the tumoral cells is related to the Breslow index, being more expressed in superficial lesions than in deepest ones and limited to the lesion. We found that all the studied markers changed their density and distribution pattern according to both TAAs expression. In particular, SCCA1^+^ cells were also Ki67^+^. SCCA1 was located both in the nuclei and in cytoplasm. Deeper lesions were also found more vascularized, as shown by the presence of a high number of vessels and more disorganized pattern. Collagen fibers showed variable type amounts, and change in their alignment.

**Conclusions:** It is important to develop new diagnostic and therapeutic strategies taking into account the entire tumor microenvironment, which consists of “master” cells i.e. tumoral cells, plus “slave” cells, i.e. stromal cells, innate and adaptive immune cells, endothelial and lymphatic cells, nervous system cells, adipocytes, and the reactive ECM. Although it has been demonstrated that the immune contexture of tumor microenvironment is able influence the course of the disease [5], our results demonstrate that the tumor microenvironment is a complex network of cells of different type that dynamically and heterogeneously in space interact each other, exchanging matter, information, and energy to advance. The understanding on these interactions might improve our knowledge on the dynamics of melanoma, and design appropriate therapeutic strategies.

**References**

1. Fox BA, Schendel DJ, Butterfield LH, Aamdal S, Allison JP, Ascierto PA, Atkins MB, Bartunkova J, Bergmann L, Berinstein N et al.: Defining the critical hurdles in cancer immunotherapy. J Trans Med. 2011;9:214.

2. Chiriva-Internati M, Grizzi F, Bright RK, Martin Kast W. Cancer immunotherapy: avoiding the road to perdition. J Trans Med. 2004;2(1):26.

3. Karagiannis P, Fittall M, Karagiannis SN: Evaluating biomarkers in melanoma. Front Oncol. 2014;4:383.

4. Roesch A. Tumor heterogeneity and plasticity as elusive drivers for resistance to MAPK pathway inhibition in melanoma. Oncogene. 2015;34(23):2951–57.

5. Galon J, Fox BA, Bifulco CB, Masucci G, Rau T, Botti G, Marincola FM, Ciliberto G, Pages F, Ascierto PA et al. Immunoscore and Immunoprofiling in cancer: an update from the melanoma and immunotherapy bridge 2015. J Trans Med. 2016;14:273.

### P9 Myeloid derived suppressor cells (MDSC) monitoring in melanoma patients treated with immunotherapy

#### Lavinia Benini^2^, Stefano Ugel^1^, Sara Cingarlini^2^, Alessandra Fiore^1^, Elisabetta Grego^2^, Giampaolo Tortora^2^, Vincenzo Bronte^1^, Luca Tondulli^2^

##### ^1^Department of Immunology, Policlinico G.B. Rossi Hospital, Verona, Italy, ^2^Department of Medical Oncology, Policlinico G.B. Rossi Hospital, Verona, Italy

###### **Correspondence:** Lavinia Benini - lavibenini@hotmail.it

*Journal of Translational Medicine* 2016, **15(Suppl 1)**:P9

**Background:** It has been thoroughly studied the relation between host immunity and development of cancer; in the scenario of immunotherapy, growing interest has been shown for MDSC, immune-suppressive myeloid cells significantly expanded in cancer patients. Strong evidences support a correlation between levels of circulating MDSC and clinical stage, outcome and response to therapy in different tumors [1, 2].

After many efforts, MDSC phenotype has been defined (currently, there are three main subsets: polymorphonuclear (PMN)-MDSC (CD11b+ CD14− CD15+), monocytic (M)-MDSC (CD11b+ CD14+ CD15−) and early-stage (e)-MDSC (Lin−(CD3/14/15/19/56)/HLA-DR−/CD33+) [3]); on the other hand, are still subject of research their immunosuppressive actions (that include aminoacid deprivation, thanks to enzymes as iNOS and ARG1).

Furthermore, it’s being investigated the use of MDSC as early marker for neoplasia and predictive marker to discriminate responder vs non-responder patients to immunotherapy [5].

**Materials and methods:** We took blood and processed it fresh from 11 patients with advanced malignant melanoma; 9 patients received immunotherapy (7 anti CTLA-4, 2 anti PD1 drugs). For eight patients were available data of pre-/post- treatment. Age-/sex-matched people were enrolled as healthy controls. MDSC characterization was performed by flow cytometry, using an antibodies-mix approved by a proficiency panel [6]. Peripheral blood mononuclear cells (PBMCs) were isolated from both patients and healthy donors; by immunomagnetic sorting we separated CD14+ cells, and analyzed *arg1* expression by RealTime-PCR.

**Results:** At 6 months, four patients were free from PD. Applying the proficiency panel designed by the Cancer Immunoguiding Program, we identified MDSC subpopulations in our patients; the frequencies of MDSC were statistically higher in all the patients compared to healthy controls. Interestingly, after treatment with check-point inhibitors MDSC levels decreased. In 5 tested patients *arg1* expression was increased at baseline and decreased in all the patients after treatment. Among our small group of patients, no correlation was found between clinical outcome and changes in the number of MDSC.

**Conclusions:** Despite the small number of patients analyzed, we demonstrated that MDSC are statistically higher in advanced melanoma patients compared to healthy controls. Their frequency significantly decreased after immunotherapy with check-point inhibitors (i.e. anti-CTLA4 and PD-1). Besides lowering the number of MDSC, therapy is able to induce also a reduction in their functional activity. In fact Arg-specific mRNA decreased during treatment.

These findings set the basis for a deeper study in melanoma patients of functional changes in MDSC at baseline and after therapy. A longer follow-up is also required in order to correlate laboratory data with clinic.

**References**

1. Idorn M, Køllgaard T, Kongsted P, Sengeløv L, Thor Straten P. Correlation between frequencies of blood monocytic myeloid-derived suppressor cells, regulatory T cells and negative prognostic markers in patients with castration-resistant metastatic prostate cancer. Cancer Immunol Immunother. 2014;63(11):1177–87. doi:10.1007/s00262-014-1591-2

2. Khaled YS, Ammori BJ, Elkord E. Increased levels of granulocytic myeloid-derived suppressor cells in peripheral blood and tumour tissue of pancreatic cancer patients. J Immunol Res. 2014;879897. doi:10.1155/2014/879897

3. Bronte V, Brandau S, Chen S, Colombo MP, Frey AB, Greten TF, Rodriguez PC. Recommendations for myeloid-derived suppressor cell nomenclature and characterization standards. Nat Commun. 2016;7:1–10. doi:10.1038/ncomms12150.

4. Gebhardt C, Sevko A, Jiang H, Lichtenberger R, Reith M, Tarnanidis K, Umansky V. Myeloid cells and related chronic inflammatory factors as novel predictive markers in melanoma treatment with ipilimumab. Clin Cancer Res. 2015;21(24):5453–9. doi:10.1158/1078-0432.CCR-15-0676

5. Mandruzzato S, Brandau S, Britten CM, Bronte V, Damuzzo V, Gouttefangeas C, Walter S. (2016). Toward harmonized phenotyping of human myeloid-derived suppressor cells by flow cytometry: results from an interim study. Cancer Immunology, Immunotherapy. doi:10.1007/s00262-015-1782-5

### P10 Prognostic value of sentinel lymph node tumor burden measurement in melanoma patients

#### Gianluca Di Monta^1^, Corrado Caracò^1^, Ugo Marone^1^, Lucia Festino^2^, Paolo A. Ascierto^2^, Nicola Mozzillo^1^

##### ^1^Dipartimento Melanoma, Tessuti molli, Muscolo-Scheletrico e Testa-Collo Chirurgia Oncologica Melanoma Istituto Nazionale Tumori “G. Pascale”, Via M. Semmola, 80131 Naples, Italy, ^2^Dipartimento Melanoma, Tessuti molli, Muscolo-Scheletrico e Testa-Collo Oncologia Medica Melanoma Immunoterapia Oncologica e Terapie Innovative Istituto Nazionale Tumori “G. Pascale”, Via M. Semmola, 80131 Naples, Italy

###### **Correspondence:** Gianluca Di Monta - gidimonta@gmail.com

*Journal of Translational Medicine* 2016, **15(Suppl 1)**:P10

**Background:** Sentinel lymph node (SLN) status is one of the most important prognostic factor in primary cutaneous melanoma patients. Patients submitted to SLN biopsy have a longer disease-free survival compared with those who are managed by delayed lymph node dissection upon eventual recurrence. To date, the best validated prognostic SN tumour burden parameters are: tumour penetrative depth (TPD) beneath the SN capsule, maximum size of SN tumour deposits and intranodal location of SN tumour. The aim of the present study was to validate the prognostic significance of various SN tumour burden micromorphometric features and their correlation with the prediction of additional lymph node metastasis, in patients with a positive sentinel node.

**Materials and methods:** In 98 SLN-positive patients treated between 2013 and 2016 at National Cancer Institute of Naples, Italy, indices of primary cutaneous melanoma and SLN tumour burden (Cochran, Starz and Rotterdam criterion) were evaluated. Multivariate analysis for the prediction of additional lymph node metastasis in patients with a positive sentinel node and with tumor burden <4% according to Cochran method was performed. Of 98 patients, 88 were submitted to complete lymph node dissection (CLND).

**Results:** CLND was positive for further lymph nodes metastases in 28 (31,8%) of total 88 cases. Among different tumor burden measurement techniques analyzed, Cochran showed a p-value of 0.02, while Starz and Rotterdam 0.3 and 0.17 respectively, for the prediction of additional lymph node metastases in patients with positive SLN, while primary tumor micromorphometric features were not statistically relevant.

**Conclusions:** In this single-center study, primary tumour and SLN tumour burden parameters have been assessed and compared to provide reliable prognostic information in SLN-positive patients. Sentinel limph node tumour deposit size <4% separated the cohort of patients, predicting negative subsequent CLND and was substantial independent predictor of NSN-positivity when >4%. According to this and other author’s recent experiences, SLN tumour burden measurement is claimed to become the most prognostic feature that recognize melanoma patients into predictive prognostic groups regarding NSN-positivity and survival outcomes.

